# Enzymatically promoted release of organic molecules linked to magnetic nanoparticles

**DOI:** 10.3762/bjnano.9.92

**Published:** 2018-03-27

**Authors:** Chiara Lambruschini, Silvia Villa, Luca Banfi, Fabio Canepa, Fabio Morana, Annalisa Relini, Paola Riani, Renata Riva, Fulvio Silvetti

**Affiliations:** 1 Department of Chemistry and Industrial Chemistry, Università di Genova, via Dodecaneso, 31 16146 Genova, Italy

**Keywords:** drug delivery, enzyme catalysis, magnetic nanoparticles, magnetic properties, peptides

## Abstract

Magnetite-based magnetic nanoparticles have been successfully coupled to an organic system constituted of a fluorescent molecule, a tripeptide specifier and a spacer. The system is able to selectively release the fluorescent molecule upon targeted enzymatic hydrolysis promoted by a lysine/arginine specific protease.

## Introduction

A major challenge of current cancer therapies is to improve the selectivity of chemotherapeutic agents against tumour cells. This goal may be achieved by exploiting smart drug delivery approaches.

Magnetic nanoparticles (NPs) [1] are a major class of nanoscale materials, which are actively investigated as carriers for targeted drug delivery [2–3]. In this approach, the nanoparticles that are carrying the appropriate drug are remotely directed to the disease site by means of a magnetic field gradient. Then the drug is typically released to the disease area through an unspecific mechanism.

Another promising drug delivery approach in cancer therapy is directed enzyme prodrug therapy (DEPT) [4–5], where a prodrug is enzymatically converted into the active form by an enzyme which is localized close to the cancer cells. To achieve selectivity, there are two main strategies. In the first one, the enzyme is exogenous and is artificially introduced into the body and selectively targeted to the tumour tissue using genes, viruses or antibodies (GDEPT, VDEPT, and ADEPT, respectively). Alternatively, the enzyme may already be present, being overexpressed by the cancerous cells themselves [6–8]. The latter approach, which is known by the acronym TAP for tumour activated prodrugs [9] or PMT for prodrug monotherapy [10], is particularly attractive due to its simplicity, not needing complex means for delivering an exogenous enzyme to the desired site.

Both the use of magnetic nanoparticles and the DEPT approach have the limitation that complete selectivity is not possible in the release of the active chemotherapeutic agent. For example, an unspecific release of the drug from the nanoparticles may take place before they have reached the desired location, while in TAP/PMT, the required enzyme may also be expressed (albeit in a lower concentration) in healthy cells.

Therefore, our idea was to combine both drug delivery approaches, achieving an enhanced selectivity. In this way, the carrier (i.e., the magnetic nanoparticle) would be directed to the tumour site, but the drug is released only when the overexpressed enzyme is present, becoming active.

However, while conjugation of enzymes onto nanoparticles (including magnetic NPs) has been often studied [11–15] (proving that the enzymatic activity is retained), very few studies have been published on the enzymatic reaction of small substrates linked to nanoparticles [16–19]. This strategy seemed indeed quite challenging due to a number of issues. The proximity of the nanoparticle may strongly influence the enzymatic activity if an appropriate spacer is not inserted. Moreover, the linker must be designed in order to be suitably attached to both the drug and the nanoparticle, and the chemistry used must be compatible with the nanoparticle. Finally, the linker must be stable under physiological conditions, avoiding unwanted release of the drug in locations different from the disease site. To our knowledge, only few examples concerning magnetic NPs have been published so far, where membrane-type matrix metalloproteases [20], cathepsin [21–22], and gelatinase [23–24] as the key drug-releasing enzymes are used.

On the basis of our previous experience in using the TAP/PMT strategy in activation of enediyne prodrugs [25–26], we decided to use a linker conceived to allow drug release by the action of a selective protease, such as plasmin. Plasmin is a serine protease that is formed upon cleavage of plasminogen by a urokinase-type plasminogen activator (u-PA), a protein associated with tumour invasion and metastasis [27–28]. This enzyme has been often used in TAP strategies [6,29–31], and the efficacy of this strategy in selective targeting of tumour cells has been demonstrated [32–33].

In this preliminary exploratory work we decided not to bind a real drug, but simply a fluorescent molecule, in order to facilitate analysis of enzymatic cleavage and obtain the first proof of concept of the enzymatic release of a small organic molecule bound to a magnetic nanoparticle.

## Results and Discussion

Magnetite nanoparticles were obtained by two different methodologies. The first one was a coprecipitation method from an aqueous solution of stoichiometric amounts of FeCl_2_·4H_2_O and FeCl_3_·6H_2_O under basic conditions [34–35]. In order to have a functional group suitable for joining the linker, these nanoparticles where functionalised by reaction with 3-aminopropyltri(ethoxy)silane (APTES) [36]. The final product was coded as NP@APTES.

We also prepared magnetic nanoparticles through the reverse micelle methodology, as described elsewhere [37]. In this case the nanoparticles obtained were silica-coated and already capped with APTES. They are here identified as NP@silica@APTES.

The morphology and chemical composition of these nanoparticles was studied using field emission scanning electron microscopy (FE-SEM) in combination with energy dispersive X-ray spectroscopy (EDXS) in addition to dynamic light scattering (DLS).

**Figure 1:**
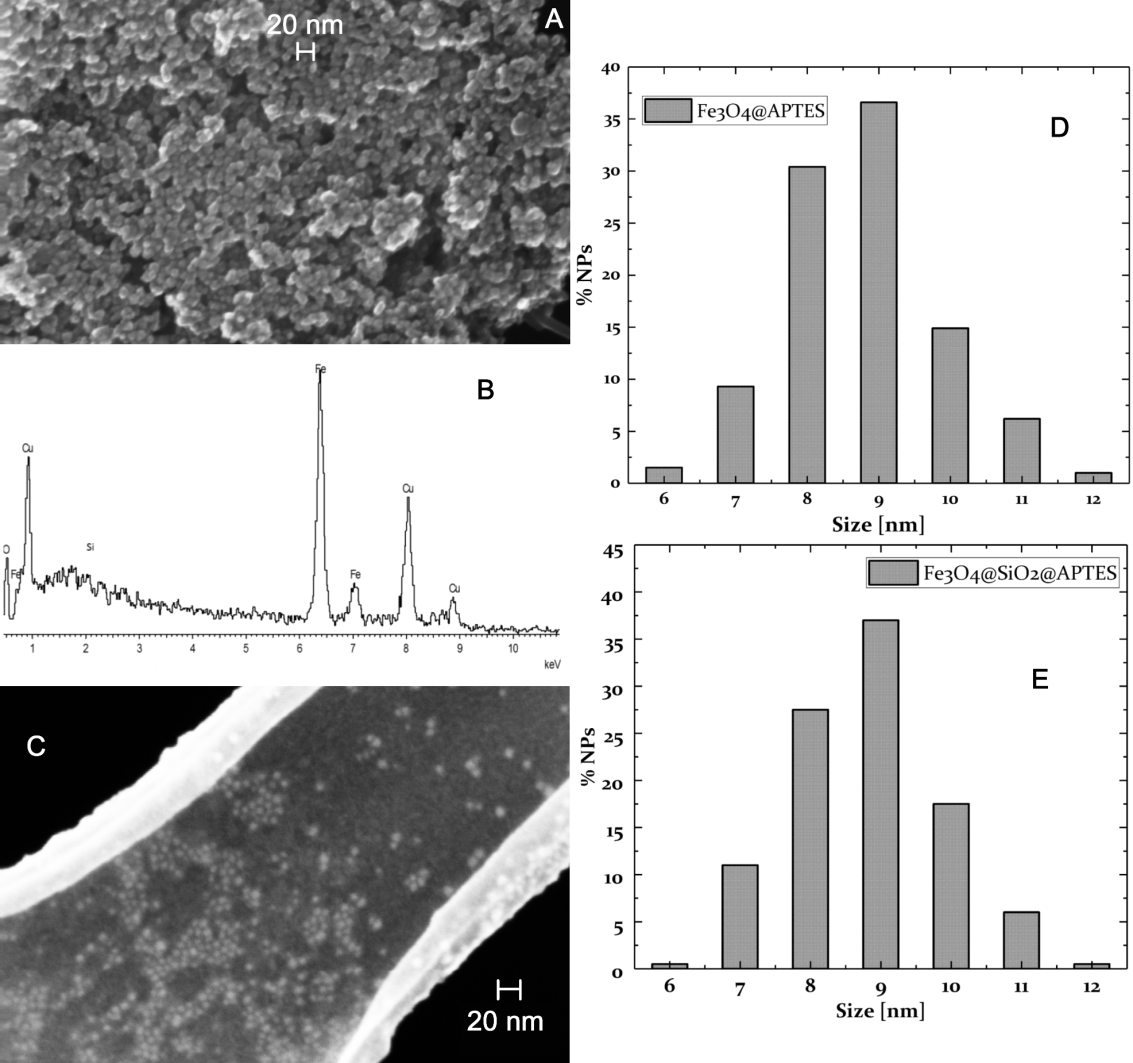
A) FE-SEM image of NP@APTES. B) EDX spectrum of NP@APTES. C) FE-SEM image of NP@silica@APTES. D) The diameter distribution of NP@APTES from ≈200 NPs. E) The diameter distribution of NP@silica@APTES from ≈200 NPs.

In Figure 1A, an FE-SEM image of NP@APTES nanoparticles is presented. The diameter distribution histogram, evaluated over 200 NPs, is also given. EDX analysis confirms the presence of the expected elements in the nanostructures, namely iron, silicon, and oxygen. The Cu and C peaks are related to the lacey carbon films of the copper grids used to deposit a drop of sample for analysis.

Due to the magnetic interactions between particles, the sample is characterized by large aggregates, which are comprised of single nanoparticles with a mean diameter of about 10 nm.

The sample NP@silica@APTES is characterized by small, spherical, uniform nanoparticles with mean diameter of about 8 nm. No large aggregates were detected.

From the DLS measurements of NP@silica@APTES samples, a peak centred at 27.7 nm (Figure S1 of Supporting Information File 1) was observed. For NP@APTES, the DLS analysis revealed larger agglomerates due to interparticle interactions where the peak was centred at 210 nm (Figure S2 in Supporting Information File 1).

As the test fluorescent molecule, we selected pyrenylmethylamine. The linker between the APTES-functionalised nanoparticles and pyrenylmethylamine can be schematically divided into two parts: a) a peptide specifier, which will act as the recognizing element for plasmin, and which will be bound to pyrenylmethylamine (or, in future, with a cytotoxic drug) through the C-terminus; b) a spacer between the peptide specifier and the nanoparticle.

On the basis of previous work by others and from our own experience, we thought that at least a tripeptide would be necessary as the peptide specifier to grant selectivity by plasmin or other similar proteases. It is well known that plasmin is selective for lysine (or, to a lesser extent, arginine) as the scissile amino acid (P_1_), while a less polar amino acid, such as leucine, is preferred at P_2_. For the P_3_ position, any amino acid is in principle suitable. However, as suggested by Katzenellenbogen et al. [38], a D-amino acid would be preferred for the amino terminus to help prevent degradation of the peptide specifier by other proteases. The choice of the spacer was not trivial, since both the peptide specifier and the APTES-functionalised nanoparticle ends with an amino group. We selected two possible ways to join these two amines: a) the transformation into an urea; or b) the coupling with a dicarboxylic acid. In the latter case, the dicarboxylic acid needs to be quite long in order to prevent intramolecular imide formation [39] with detachment of the peptide specifier from the nanoparticle.

**Scheme 1:**
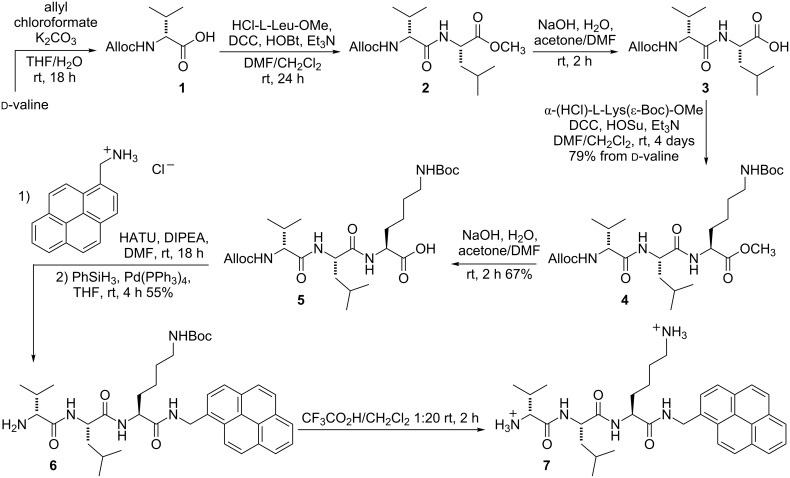
Synthesis of peptide specifier. Abbreviations: DCC – dicyclohexylcarbodiimide; HOBT – 1-hydroxybenzotriazole; HOSu – *N*-hydroxysuccinimide; DIPEA – *N*,*N*-diisopropylethylamine; HATU – 1-[bis(dimethylamino)methylene]-1*H*-1,2,3-triazolo[4,5-*b*]pyridinium 3-oxid hexafluorophosphate.

Scheme 1 reports the synthesis of the tripeptide specifier. For our purposes we needed two orthogonal protections for the D-valine and the ε-lysine amino groups. Particularly crucial is the latter, since it was planned to be removed as the last step after linking to the nanoparticles. We selected *tert*-butyloxycarbonyl (Boc) thanks to its easy removal that releases no side products. Moreover, we chose to perform the synthesis from left to right, contrary to what is typically done. The synthesis from right to left would have required a third orthogonal protection for the amino group, and the use of the fluorenylmethyloxycarbonyl (Fmoc) group proved to be rather troublesome for a solution-phase synthesis [25]. Performing the synthesis from left to right, we selected the allyloxycarbonyl (Alloc) as the second protection.

D-valine was smoothly protected as allyloxycarbamate under Schotten–Baumann conditions and then coupled with L-leucine methyl ester hydrochloride using dicyclohexylcarbodiimide (DCC) and 1-hydroxybenzotriazole (HOBt). The resulting dipeptide methyl ester was hydrolysed under basic conditions and coupled with *N*ε-Boc-L-lysine methyl ester hydrochloride using DCC and *N*-hydroxysuccinimide (HOSu), affording compound **4** with excellent yield from the starting amino acid. No racemization was detected in this latter coupling.

After hydrolysis, coupling of carboxylic acid **5** with pyrenylmethylamine was more troublesome from the stereochemical point of view. After testing several coupling agents and bases using benzylamine as the model compound (see Supporting Information File 1), we found out that the best one was 1-[bis(dimethylamino)methylene]-1*H*-1,2,3-triazolo[4,5-*b*]pyridinium 3-oxid hexafluorophosphate (HATU) in combination with *N*,*N*,-diisopropylethylamine (DIPEA) in DMF. The crude-coupled product was directly deprotected at the *N*-terminus without intermediate isolation.

This deblocking step was, not unexpectedly, problematic. Optimization was carried out on the benzyl ester of **3**. Different solvents (THF and DCM) and scavengers (pyrrolidine, PhSiH_3_, thioanisole, dimedone and triethylammonium formate) were investigated maintaining Pd(PPh_3_)_4_ as the source of Pd(0). We eventually found that the combination of a high excess of PhSiH_3_ and THF as solvent were the best conditions. The optimized conditions were then applied to the real system, affording **6** in 55% yield over two steps. The moderate yield was mainly due to the high insolubility of all pyrene-containing compounds in most organic solvents, leading to the loss of material during the workup and purification. Preliminary experiments of conjugation with Fe_3_O_4_ nanoparticles functionalized with 3-aminopropyltriethoxysilane (APTES) showed that the purification of **6** was essential. In fact, the presence of excess PhSiH_3_ and the residues of Pd were detrimental for the conjugation reaction.

Compound **6** was also deblocked at the ε-lysine amino group to provide diamine **7**, which was used as a model for the enzymatic reaction and for assessing analytical detection of the liberated fluorescent amine (see below).

**Scheme 2:**
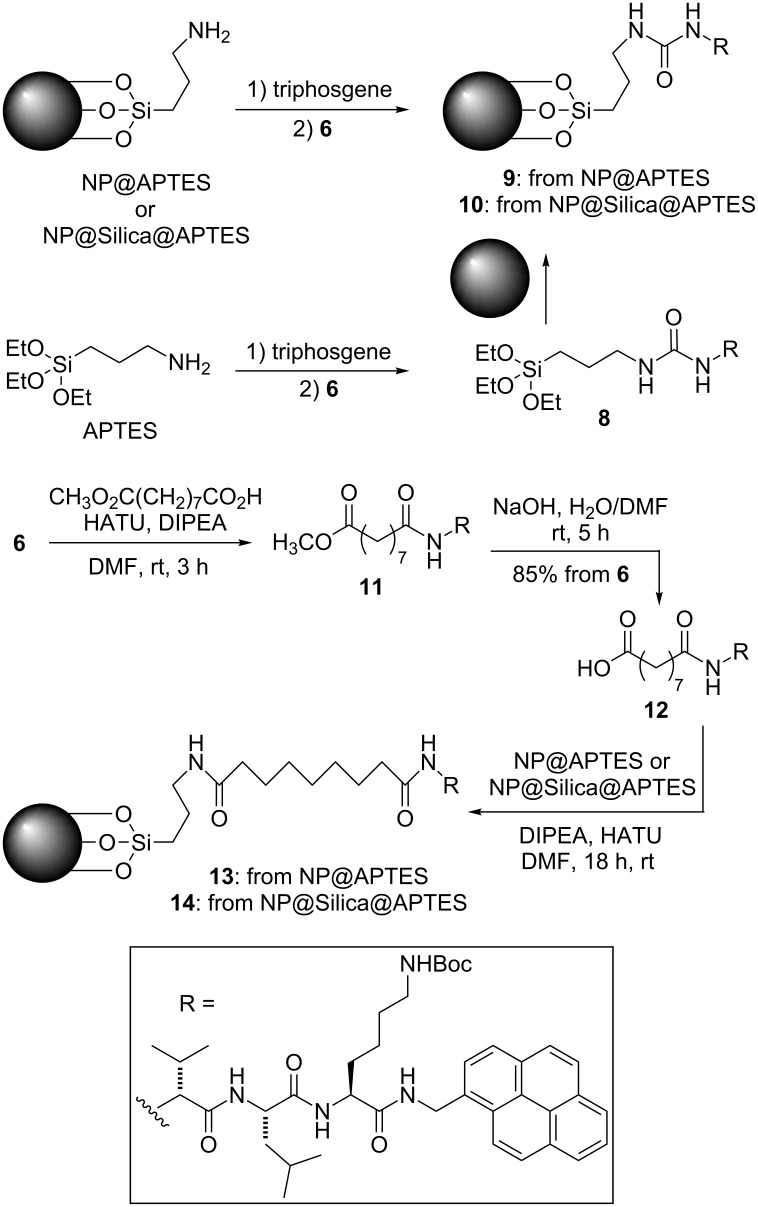
Strategies employed for linking tripeptide **6** to magnetic nanoparticles.

Scheme 2 shows the different strategies investigated for binding tripeptide **6** to the nanoparticles. We first chose urea as the linking moiety. The transformation of **6** into an isocyanate was not possible, and thus we decided to form an isocyanate from the APTES amino group. Two alternative approaches were followed, depending on when this conversion was carried out: either before or after binding of APTES to the nanoparticles. They were both investigated using NP@APTES nanoparticles. However, only the first approach was successful. When we tried to derivatize the nanoparticles with the preformed urea **8**, no loading was detected. Thus, the synthesis of **9** could be only carried out by converting the APTES-functionalised nanoparticles into an isocyanate first, by reaction with triphosgene, followed by addition of tripeptide **6**. When we tried to apply the same conditions for converting NP@silica@APTES into **10**, no loading was detected, probably because this type of functionalized NPs is too small to load an appreciable quantity of **6**; moreover, they could be more sensitive to the harsh reaction conditions. Thus, the urea spacer was viable only for the first type of nanoparticles.

In order to insert a longer spacer, and also to employ a milder methodology for conjugation of the tripeptide with the nanoparticles, we also converted tripeptide **6** into the amide **11** by coupling it with the monoester of azelaic (nonanedioic) acid. After saponification, the acid **12** was coupled with the functionalised nanoparticles. In this case, the strategy was successful for both types of nanoparticles. However, the NP@silica@APTES derived conjugate **14** was later found to be unstable to the Boc deblocking conditions, which led to destruction of the nanoparticles. Thus, we decided to concentrate our studies on the more robust NP@APTES derived conjugates.

**Figure 2:**
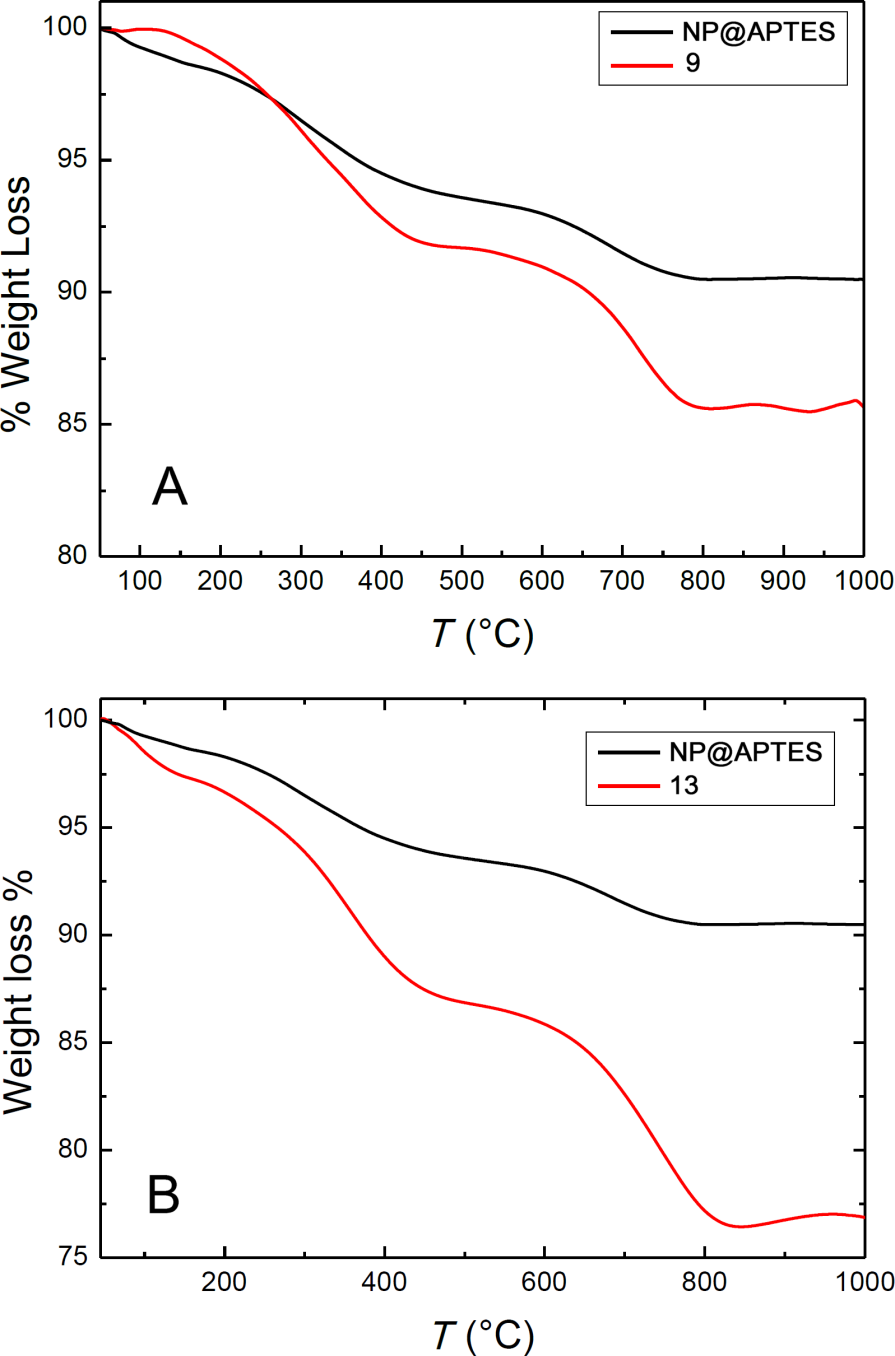
Thermogravimetric analysis profiles for precursor NP@APTES nanoparticles and for conjugate **9** (A) and **13** (B).

The relative quantity of APTES incorporated into the NPs and the loaded amount of **6** or **12** into **9** and **13** was determined by thermogravimetric analysis (TGA) (Figure 2). The amount of APTES resulted to be 9.5%. TGA results for **9** and **13** showed a weight loss of 14.3% and 23.5%, respectively. Considering the initial amount of APTES, the loading of **6** and **11** onto the NPs was found to be 5.3% (corresponding to 79 μmol/g of material) and 15.5% (corresponding to 184 μmol/g of material), respectively. Thus, the azelate linker allows a more efficient loading (about double) than the urea linker.

**Figure 3:**
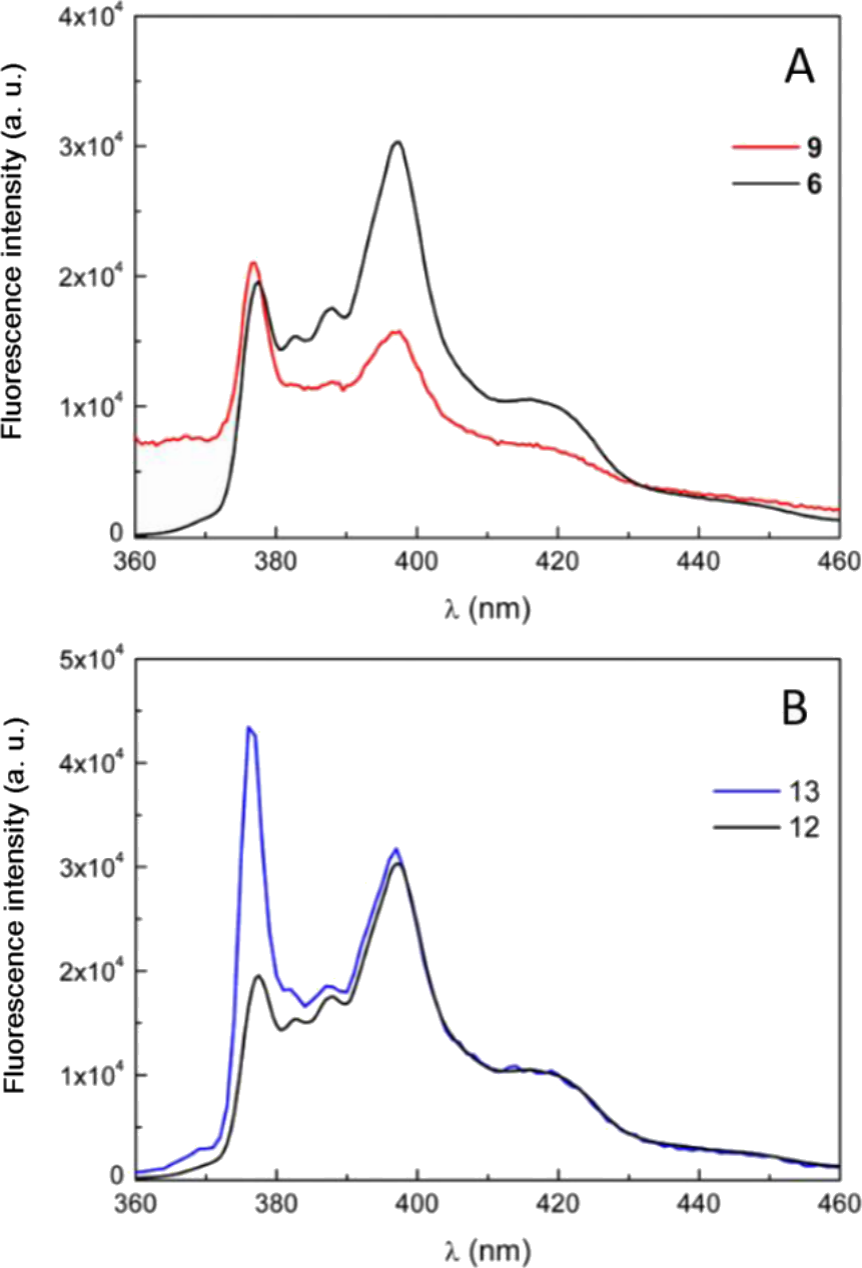
A) Fluorescence spectra of **6** (black curve) and **9** (red curve). B) Fluorescence spectra of **12** (black curve) and **13** (blue curve).

Figure 3A shows the fluorescence spectra measured on the unbound amine **6** and on the conjugated system **9** using an excitation wavelength of 345 nm. All spectra were recorded using a DMSO solution of the samples. No fluorescence signal was detected for the APTES-coated magnetic NPs.

The spectrum of **6** is similar to the fluorescent spectrum of pyrene. The fluorescence emission spectrum of pyrene, and therefore of **6**, is characterized by an ensemble of four major bands with well-defined maxima at ≈375, 388, 398, and 415 nm, respectively.

The peaks are attributed to the π → π* transitions and are cumulatively defined as monomeric emission. The peak at 375 nm corresponds to the first vibronic band with a 0–0 transition, while the one at 388 nm is attributed to the third vibronic band with a 0–2 transition.

The coupling reaction of **6** with NP@APTES causes a slightly different emission profile composed by all the peaks detected for free **6** but with different intensity, especially for band I (378 nm) and III (398 nm). This evidence can be ascribed to the effective coupling that occurs on the surface of the nanoparticles that affects the mobility, forcing the molecule in fixed conformations.

A similar behaviour is observed with the azelate-linked conjugate **13**. Figure 3B reports the fluorescence spectra for this compound and for unconjugated **12**.

**Figure 4:**
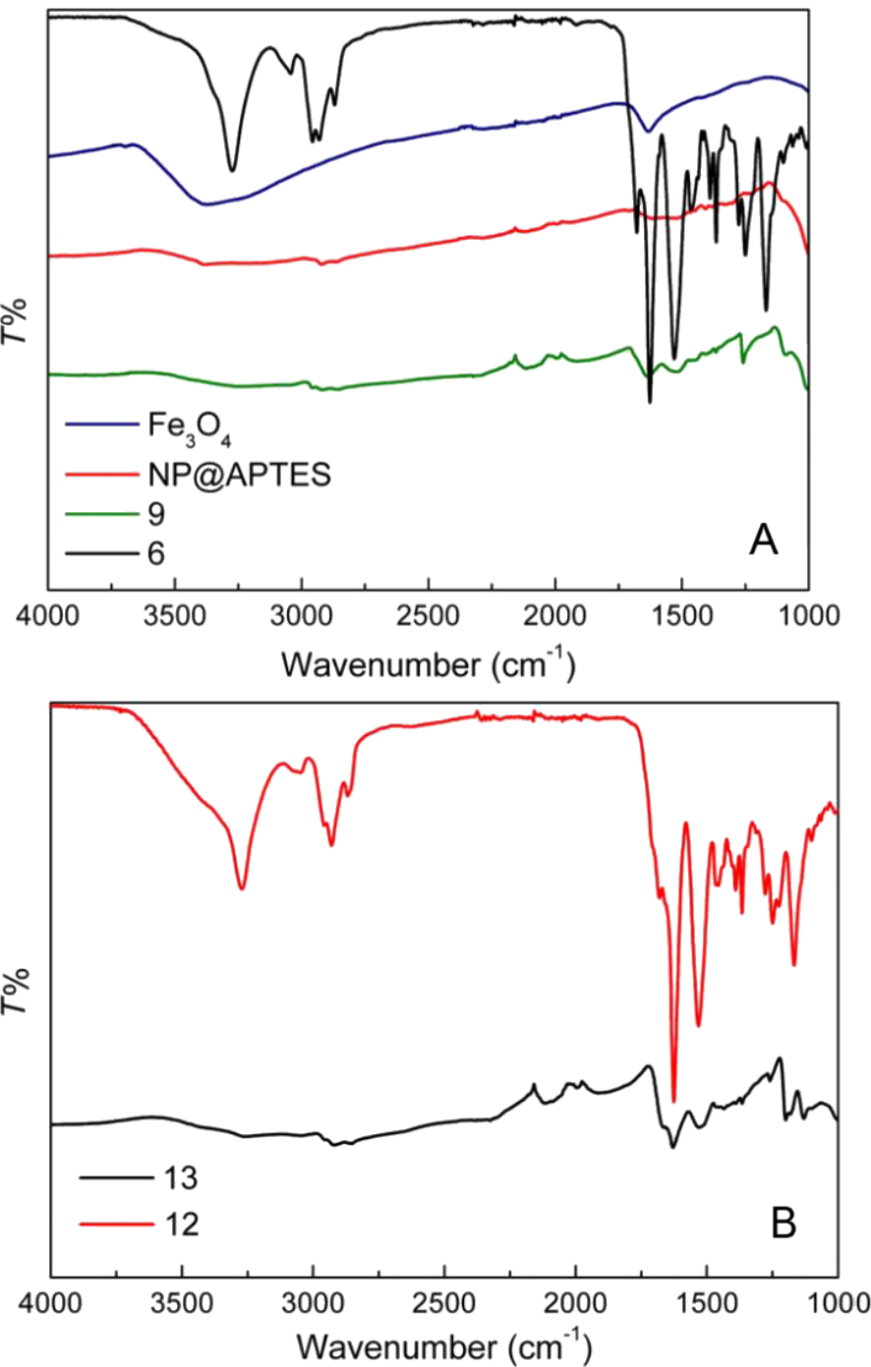
A) Infrared transmission spectra of **9** compared with nonconjugated **6** and with NP@APTES and magnetite; B) infrared transmission spectra of **13** compared with nonconjugated **12**.

Finally, the infrared spectra of both **9** and **13** are reported in Figure 4 and compared with the spectra of NP@APTES and of magnetite. Although a broadening of the peaks is observed, the signals characteristic of the tripeptide, the linker and pyrene, are also present in the conjugated NPs.

In particular, signals related to carbonyl stretching, deriving from **6** and **12**, can be observed at 1650 cm^−1^ in both conjugated samples, **9** and **13**.

Then we turned our attention to the enzymatic cleavage of the fluorophore from the tripeptide. In order to check the affinity of our peptide, and to select the correct amount of enzyme to be used, we carried out some experiments with model compound **7**, using trypsin and plasmin as proteases. Trypsin, like plasmin, has a preference for lysine (or arginine) as the scissile (P_1_) amino acid. The kinetic of the hydrolysis was studied by the HPLC method with fluorescence detection (HPLC-FLD). The results showed that both enzymes recognized the substrate and after 72 h at 37 °C the conversion was complete. In particular, 0.023 U of plasmin were able to fully release pyrenylmethylamine from 50 nmol of **7** in 72 h. The conversion was already 88% after 24 h. Trypsin displayed a similar behaviour. The units for this enzyme were not provided, but comparing the rates, we established that 170 mg of trypsin had the same catalytic efficiency as 1 U of plasmin. Thus, reaction on 50 nmol of **7** was complete in 48 h using 4.6 μg of trypsin. In both cases, the kinetics was found to be first order with respect to the substrate. Since the aim of our work was mainly to check the compatibility of the nanoparticles with the enzymatic reaction, the more available trypsin was used in the experiments on conjugated NPs, also taking into account the recent report by Koch et al., who showed that trypsin and plasmin had a similar behaviour on an enzymatic cleavable linker similar to ours [32].

HPLC-FLD was obviously not suited for following the enzymatic reaction of the nanoparticles. Thus, we generated a calibration curve to quantify the released pyrenylmethylamine through HPLC with a variable wavelength detector (HPLC-VWD) (see Supporting Information File 1).

First, the Boc protecting group was removed with trifluoroacetic acid/CH_2_Cl_2_. Then the two types of nanoparticles (NP@APTES with different spacers) were subjected to the enzymatic hydrolysis using a ratio of trypsin/substrate similar to that used on **7** (more precisely 123 μg/μmol and 136 μg/μmol for **9** and **13**, respectively, compared to 92 μg/μmol used for **7**). We preferred not to monitor the amount of cleavage versus time, because sampling could lead to errors due to the heterogeneity of the mixture. Thus, after 72 h at 37 °C, the mixtures were washed several times with MeOH and the washings were diluted to a precise volume. By comparison with a calibration curve, the sample injected into the HPLC-VWD allowed the liberated μmols of pyrenylmethylamine to be determined.

From these data, and from the loading determined by TGA, we calculated the conversions of the enzymatic reactions, which were 7.1% and 5.8% for the urea spacer and for the azelate spacer, respectively. Thus, although we have demonstrated that the enzymatic reaction was actually possible when the tripeptide specifier is anchored to magnetic NPs, the reaction rate is considerably lower. Clearly, the presence of the nanoparticles influences the enzymatic activity. We think that the length and nature of the spacer is of great importance in affecting the reactivity. Although we guessed that the longer azelate spacer should have produced a higher rate, our experimental evidence shows that the shorter urea spacer was even better from this point of view. The lipophilic nature of the longer spacer may have elicited an aggregation phenomena that may have made access to the active site more difficult. We should also bear in mind that with the azelate spacer the loading was higher.

**Figure 5:**
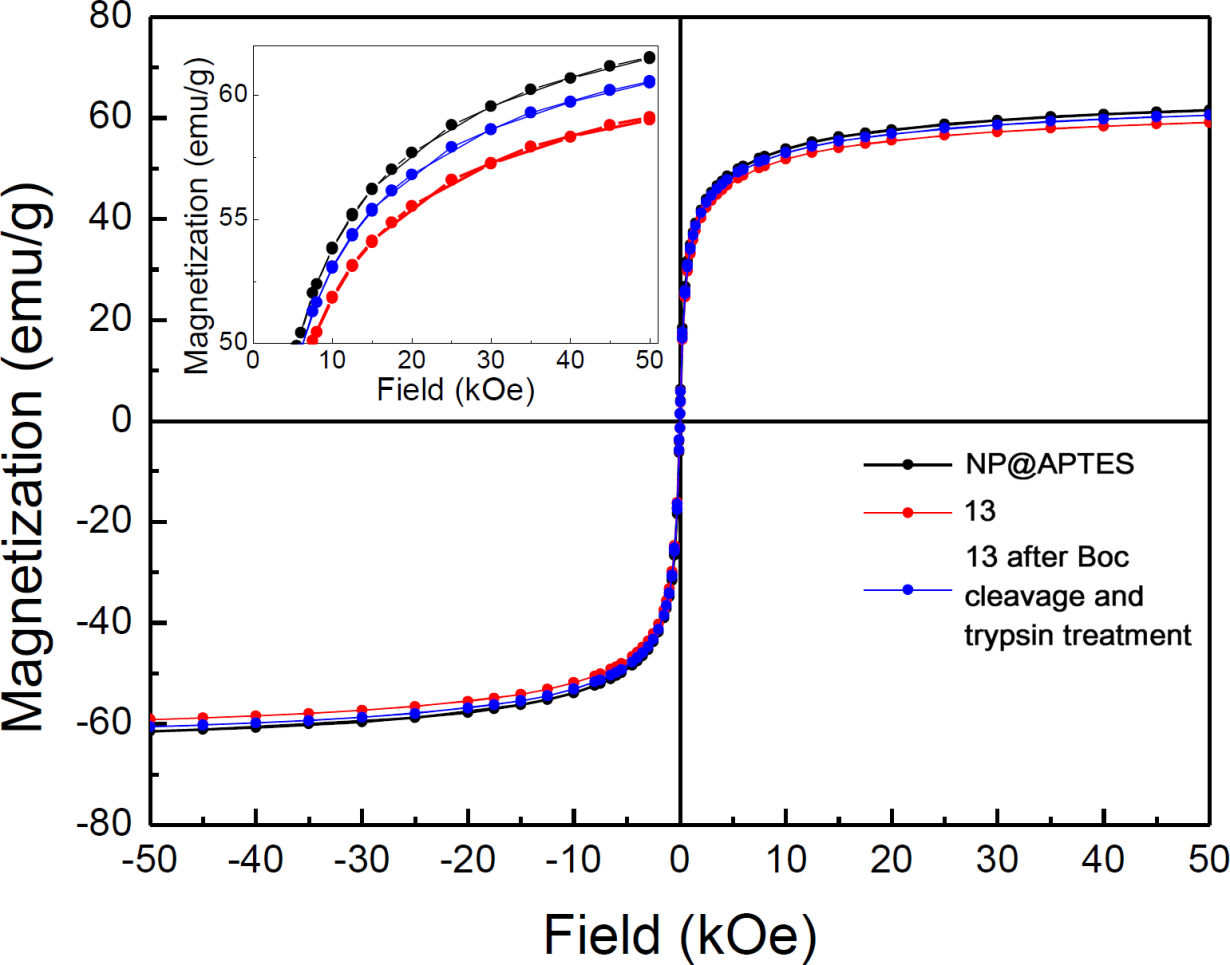
Room temperature magnetic hysteresis cycle for NP@APTES, the azelate conjugated nanoparticles (**13**) and **13** after Boc cleavage and trypsin treatment. In the inset the saturation magnetization in an enlarged scale is shown.

It is also important to assess if the magnetic properties of the nanoparticles are affected by conjugation and/or by the enzymatic reaction. The magnetic properties were investigated by measuring the hysteresis cycles at 300 K. In particular, we examined the nanoparticles NP@APTES alone and the conjugates **13** (with the azelate spacer) before and after the Boc deblocking and the enzymatic cleavage (Figure 5). Saturation magnetization values of about 60 emu/g were observed for all the samples, confirming that the material was not degraded in the coupling step, as well as during Boc deblocking and under the enzymatic hydrolysis conditions. These conjugated nanoparticles proved to be stable for two months in the freezer, since the magnetic properties and infrared spectra showed no visible changes.

## Conclusion

We have successfully demonstrated the possibility to exploit a selective protease-mediated release of an organic molecule from a magnetic nanoparticle. Although in this preliminary investigation the released molecule was only a simple fluorescent substance (pyrenylmethylamine), the same strategy can be applied to the release of other substances, including cytotoxic drugs. The tripeptide specifier has been designed in order to selectively release the organic molecule upon the action of a lysine/arginine-selective serine protease, such as trypsin or plasmin. Although the rate of enzymatic cleavage is significantly lower than that determined for the unbound tripeptide, this is not a disadvantage in view of continuous, slow release of a drug from the nanoparticle. The well-established possibility to guide magnetic nanoparticles to the malignant tissues coupled with the overexpression of proteases such as plasmin in many tumour cells might allow a substantial increase in the therapeutic index.

## Experimental

**General remarks:** All non-aqueous reactions were performed under an inert atmosphere of argon or nitrogen. Analytical thin layer chromatography was performed using F254 0.25 mm thin layer chromatography (TLC) glass plates and visualized by ultraviolet light (UV, 254 nm and 365 nm), or stained with cerium ammonium molybdate (CAM, Hanessian’s stain) or with ninhydrin or with concentrated HBr followed by ninhydrin. Chromatographic purification was performed as flash chromatography on 40–63 μm silica. Abbreviations for solvents are: dichloromethane (DCM), dimethyl sulfoxide (DMSO), petroleum ether 40-60 (PE). NMR spectra were taken at rt in *d*_6_-DMSO at 300 MHz (^1^H), and 75 MHz (^13^C), using the central peak of DMSO (^1^H 2.506 ppm, ^13^C 39.43 ppm) as the internal standard. The chemical shifts are reported in ppm (δ-scale). The peak assignments were made with the aid of gCOSY, TOCSY, gHSQC and gHMBC experiments. For high-resolution mass spectroscopy (HRMS), the samples were analysed with a Synapt G2 QToF mass spectrometer. MS signals were acquired from 50 to 1200 *m*/*z* in ESI positive ionization mode. Optical rotations were measured on a digital polarimeter at 589 nm. The [α] unit is mL·g^−1^·dm^−1^ and *c* (concentration) unit is g in 100 mL. Fourier transform infrared (FT-IR) spectra were recorded on a Perkin Elmer Spectrum 65 (Perkin Elmer, Waltham, MA, USA) instrument, equipped with a universal attenuated total reflectance (ATR) sampling accessory. The morphology of the particles was analysed using a field emission scanning electron microscope (FE-SEM, ZEISS SUPRA 40VP), collecting the signal (secondary electrons) by means of an in-lens detector; the particle microanalyses were performed with an energy dispersive X-ray spectrometer (EDXS, Oxford, INCA Energie 450 × 3). The analyses were performed collecting the signal by means of the in-lens detector. The average size of the particles was calculated by counting a minimum of 100 particles using the ImageJ software. The samples were suspended in ethanol, exposed to ultrasonic vibrations to decrease the aggregation, and deposited on a lacey carbon copper grid.

TGA was performed using a Labsys EVO Setaram instrument. Approximately 5 mg of sample was weighed in an open alumina crucible and heated from 50 °C to 1000 °C in He flux (20 mL/min) with a heating rate equal to 10 °C/min. The fluorescence spectra were acquired between 350 and 500 nm (λ_ex_ = 345 nm) at 25 °C at a concentration of NPs of 0.16 mg/mL. A Fluorolog spectrofluorometer (Horiba Jobin-Yvon, Edison, NJ) and 10 mm path length quartz cells were used. DC magnetization was performed in a dc-superconducting quantum interference device (SQUID) magnetometer (Magnetic Properties Measurement System, Quantum Design) with resolution better than 10^−7^ emu. The room temperature magnetic hysteresis cycles were obtained in the 0–5 Tesla μ_0_H magnetic field range. DLS measurements were performed using a Zetasizer Nano ZS90 instrument (Malvern Instruments, UK ). The measurements parameters were as follows: scattering angle of 90°, measurement temperature of 20 °C, ethanol as dispersant (20 °C dynamic viscosity 1.23 mPa·s, refractive index 1.3617). DLS studies were carried out in general purpose mode (normal resolution). The results (obtained from a set of three measurements for both NP@APTES (Figure S1, Supporting Information File 1) and NP@silica@APTES (Figure S2, Supporting Information File 1) are reported.

**Methyl *N*^2^-((allyloxy)carbonyl)-D-valyl-L-leucyl-*N*^2^-(*tert*-butoxycarbonyl)-L-lysinate 4:** To a solution of D-valine (3.00 g, 25.6 mmol) in 1:1 THF/H_2_O (116 mL, 0.2 M), K_2_CO_3_ (5.31 g, 38.4 mmol) was added. The mixture was cooled down at 0 °C and allyl chloroformate (3.3 mL, 30.7 mmol) was added dropwise. After stirring at rt for 18 h, the volatile components were removed and the residue was partitioned between DCM (50 mL) and H_2_O (acidified with 37% HCl to pH 2). The aqueous phase was extracted with DCM (3 × 20 mL) and the combined organic phases were washed with brine. The organic phase was dried over sodium sulfate, filtered and concentrated. The residue (pale yellow oil), corresponding to (allyloxycarbonyl)-D-valine **1**, was used in the next step without further purification. It was taken up in dry DMF (40 mL, 0.6 M), and treated in sequence with Et_3_N (3.6 mL, 25.6 mmol), L-leucine methyl ester hydrochloride (4.65 g, 25.6 mmol), and 1-hydroxybenzotriazole (3.46 g, 25.6 mmol) at 0 °C under N_2_ atmosphere. Then, a solution of dicyclohexylcarbodiimide (4.81 g, 28.2 mmol) in dry DCM (15 mL, 0.2 M) was added at 0 °C under N_2_ atmosphere. After stirring at 0 °C for 1 h and at rt for 24 h, DCM (15 mL) was added and the reaction mixture was kept at −20 °C overnight. The white solid was filtered off and the solution was partitioned between DCM and H_2_O (50 mL). The aqueous phase was extracted with DCM (2 × 20 mL) and the combined organic phases were washed with NH_4_Cl (saturated solution), NaHCO_3_ (saturated solution) and brine. The organic phase was dried over sodium sulfate, filtered and concentrated to give crude **2** as a white foam, which was used as such in the next step without further purification. It was taken up in acetone (70 mL) and DMF (30 mL) and treated, dropwise at rt, with 1 M aqueous NaOH (51 mL, 51.2 mmol). After stirring for 2 h, the volatile components were removed and the residue was partitioned between EtOAc (50 mL) and H_2_O (50 mL, acidified with 37% HCl until pH 2). The aqueous phase was extracted with EtOAc (3 × 20 mL) and the combined organic phases were washed with brine. The organic phase was dried over sodium sulfate, filtered and concentrated to give crude acid **3** (pale-yellow foam) (8.45 g), which was used in the next step without further purification. An aliquot of **3** (1.041 g, corresponding to theoretical 3.15 mmol) was taken up in dry DMF (10 mL, 0.3 M) and treated with Et_3_N (460 μL, 3.31 mmol), *N*ε-Boc-L-lysine methyl ester hydrochloride (893 mg, 3.31 mmol) and *N*-hydroxysuccinimide (495 mg, 4.30 mmol) at rt under N_2_ atmosphere. After 15 min, a solution of dicyclohexylcarbodiimide (887 mg, 4.30 mmol) in dry DCM (5 mL, 0.9 M) was added at 0 °C under N_2_ atmosphere. After stirring at rt for 4 days, EtOAc (10 mL) was added and the reaction mixture was kept at −20 °C overnight. The white solid was filtered off and the solution was partitioned between EtOAc (20 mL) and NaHCO_3_ (saturated solution, 30 mL). The aqueous phase was extracted with EtOAc (3 × 20 mL) and the combined organic phases were washed with 5% (NH_4_)H_2_PO_4_ (aqueous solution) and brine. The organic phase was dried over sodium sulfate, filtered and concentrated. The residue was purified by flash column chromatography on silica gel eluting with 40% EtOAc in petroleum ether + 1% EtOH to give **4** (1.38 g, white foam, 79% from D-valine). *R*_f_ 0.32 (PE/EtOAc 6:4 + 1% EtOH; HBr followed by ninhydrin). [α]_D_^20^ −17.8 (*c* 1.0, CHCl_3_); ^1^H NMR (300 MHz, DMSO-*d*_6_, 25 °C) δ 8.15 (d, ^3^*J*_H,H_ = 8.3 Hz, 1H, NH Leu), 8.10 (d, ^3^*J*_H,H_ = 7.3 Hz, 1H, NH Lys), 7.26 (d, ^3^*J*_H,H_ = 8.3 Hz, 1H, NH Alloc), 6.75 (t, ^3^*J*_H,H_ = 5.6 Hz, 1H, NH Boc), 6.00–5.77 (m, 1H, CH_2_=C*H*CH_2_O), 5.28 (dd, ^3^*J*_H,H_ = 17.3 Hz, ^2^*J*_H,H_ = 1.8 Hz, 1H, C*H*H=CHCH_2_O), 5.16 (dd, ^3^*J*_H,H_ = 10.4 Hz, ^2^*J*_H,H_ = 1.6 Hz, 1H, C*H*H=CHCH_2_O), 4.50–4.40 (m, 2H, CH_2_=CHC*H*_2_O), 4.31 (q, ^3^*J*_H,H_ = 7.9 Hz, 1H, α-CH Leu), 4.22–4.10 (m, 1H, α-CH Lys), 3.82 (t, ^3^*J*_H,H_ = 7.9 Hz, 1H, α-CH Val), 3.58 (s, 3H, OCH_3_), 2.88 (q, ^3^*J*_H,H_ = 6.4 Hz, 2H, ε-CH_2_ Lys), 2.01–1.82 (m, 1H, β-CH Val), 1.79–1.52 (m, 3H, α-CH_2_ Lys + γ-CH Leu), 1.52–1.41 (m, 2H, β-CH_2_ Leu), 1.41–1.18 (m, 13H, *t*Bu + γ-CH_2_ Lys + δ-CH_2_ Lys), 0.91–0.79 (m, 12H, 4×CH_3_ Val and Leu); ^13^C NMR (75 MHz, DMSO-*d*_6_, 25 °C) δ 172.4 (C=O), 172.3 (C=O), 171.2 (C=O), 156.1 (Alloc C=O), 155.6 (Boc C=O), 133.6 (CH_2_=*C*HCH_2_O), 117.0 (*C*H_2_=CHCH_2_O), 77.4 (*t-*Bu C quat.), 64.5 (CH_2_=CH*C*H_2_O), 60.5 (α-CH Val), 52.0 (α-CH Lys), 51.7 (OCH_3_), 50.6 (α-CH Leu), 40.4 (β-CH_2_ Leu), 39.5 (ε-CH_2_ Lys), 30.4 (β-CH_2_ Lys), 30.0 (β-CH Val), 29.1 (CH_2_ Lys), 28.3 (*t-*Bu CH_3_), 24.1 (γ-CH Leu), 23.2 (CH_3_), 22.8 (CH_2_ Lys), 21.2 (CH_3_), 19.1 (CH_3_), 18.3 (CH_3_); IR (KBr) 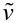: 3296 (w), 3076 (w), 2958 (w), 2871 (w), 1731 (w), 1682 (m), 1638 (s), 1522 (s), 1463 (w), 1389 (w), 1366 (m), 1343 (w), 1269 (m), 1245 (m), 1169 (m), 1129 (m), 1040 (m), 1016 (m), 993 (w), 926 (w), 867 (w), 778 (w) cm^−1^; HRMS (ESI^+^) *m*/*z*: [M + H^+^] calcd for C_27_H_49_N_4_O_8_: 557.3550; found: 557.3551.

***N*^2^-((Allyloxy)carbonyl)-D-valyl-L-leucyl-*N*^6^-(*tert*-butoxycarbonyl)-L-lysine (5):** To a solution of **4** (1.30 g, 2.34 mmol) in 1:2.5 DMF/acetone (15 mL, 0.16 M), 1 M NaOH (aqueous solution, 4.8 mL, 4.80 mmol) was added at rt. After stirring for 2 h, the volatile components were removed and the residue was partitioned between EtOAc (30 mL) and H_2_O (40 mL, acidified with 37% HCl until pH 2). The aqueous phase was extracted with EtOAc (3 × 20 mL) and the combined organic phases were washed with brine (3×). The organic phase was dried over sodium sulfate, filtered and concentrated. The residue was purified by flash column chromatography on silica gel eluting with 5% MeOH in DCM + 1% AcOH to give **5** (847 mg, white foam, 67%, AcOH removed as azeotrope with heptane). *R*_f_ 0.25 (DCM/MeOH 95:5 + 1% AcOH; HBr followed by ninhydrin). [α]_D_^20^ −8.61 (*c* 1.0, CHCl_3_); ^1^H NMR (300 MHz, DMSO-*d*_6_, 25 °C) δ 12.46 (brs, 1H, COOH), 8.14 (d, ^3^*J*_H,H_ = 8.3 Hz, 1H, NH Leu), 7.98 (d, ^3^*J*_H,H_ 7.6 Hz, 1H, NH Lys), 7.25 (d, ^3^*J*_H,H_ = 8.4 Hz, 1H, NH Alloc), 6.76 (t, ^3^*J*_H,H_ 5.4 Hz, 1H, NH Boc), 6.04–5.76 (m, 1H, CH_2_=C*H*CH_2_O), 5.28 (dd, ^3^*J*_H,H_ =17.2 Hz, ^2^*J*_H,H_ = 1.6 Hz, 1H, C*H*H=CHCH_2_O), 5.16 (dd, ^3^*J*_H,H_ = 10.4 Hz, ^2^*J*_H,H_ = 1.4 Hz, 1H, C*H*H=CHCH_2_O), 4.50–4.41 (m, 2H, CH_2_=CHC*H*_2_O), 4.37–4.25 (m, 1H, α-CH Leu), 4.14–4.02 (m, 1H, α-CH Lys), 3.83 (t, ^3^*J*_H,H_ = 7.8 Hz, 1H, α-CH Val), 2.88 (q, ^3^*J*_H,H_ = 6.3 Hz, 2H, ε-CH_2_ Lys), 2.00–1.83 (m, 1H, β-CH Val), 1.77–1.17 (m, 18H, *t*Bu + β-CH_2_ Leu + γ-CH Leu + β-CH_2_ Lys + γ-CH_2_ Lys + δ-CH_2_ Lys), 0.93–0.76 (m, 12H, 4×CH_3_ Val and Leu); ^13^C NMR (75 MHz, DMSO-*d*_6_, 25 °C) δ 173.4 (C=O COOH), 172.0 (C=O Leu), 171.2 (C=O Val), 156.1 (C=O Alloc), 155.5 (C=O Boc), 133.6 (CH_2_=*C*HCH_2_O), 117.0 (*C*H_2_=CHCH_2_O), 77.3 (*t-*Bu C quat.), 64.5 (CH_2_=CH*C*H_2_O), 60.5 (α-CH Val), 52.00 (α-CH Lys), 50.5 (α-CH Leu), 40.5 (α-CH_2_ Leu), 39.7 (ε-CH_2_ Lys), 30.6 (CH_2_ Lys), 30.1 (β-CH Val), 29.1 (CH_2_ Lys), 28.3 (*t-*Bu CH_3_), 24.1 (γ-CH Leu), 23.2 (CH_3_), 22.9 (CH_2_ Lys), 21.1 (CH_3_), 19.1 (CH_3_), 18.3 (CH_3_) ppm; IR (KBr) 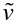: 3297 (w), 2961 (w), 2873 (w), 1709 (m), 1645 (m), 1526 (m), 1454 (w), 1392 (m), 1367 (m), 1246 (m), 1167 (m), 1036 (w), 994 (w), 929 (w), 861 (w), 777 (w), 736 (w), 668 (m), 607 (m) cm^−1^; HRMS (ESI^+^) *m*/*z* [M + H^+^]: calcd for C_26_H_47_N_4_O_8_: 543.3394; found: 543.3398.

***tert*-Butyl ((*S*)-5-((*S*)-2-((*R*)-2-amino-3-methylbutanamido)-4-methylpentanamido)-6-oxo-6-((pyren-1-ylmethyl)amino)hexyl)carbamate (6):** A suspension of 1-pyrenemethylamine hydrochloride (197 mg, 0.737 mmol) in dry DMF (25 mL, 0.03 M) was treated with DIPEA (642 μL, 3.68 mmol), peptide **5** (400 mg, 0.737 mmol) and HATU (280 mg, 0.737 mmol) at rt under N_2_ atmosphere. After stirring at rt for 18 h, the mixture was partitioned between EtOAc (40 mL) and brine (40 mL). Although the desired product was rather insoluble in both phases, it tends to disperse in the organic phase, and thus separation was anyway possible. The phases were separated and the aqueous phase was re-extracted twice with EtOAc (2 × 20 mL). The combined organic phases were washed with brine (3×) and concentrated to dryness. The residue (yellow solid) was used in the next step without further purification. It was suspended in dry and degassed THF (14 mL, 0.05 M) and treated with Pd(PPh_3_)_4_ (85 mg, 10 mol %) and phenylsilane (910 μL, 7.37 mmol) at 0 °C under an Ar atmosphere. After stirring at rt for 4 h, the dark mixture was concentrated and purified by flash column chromatography on silica gel eluting with 5% MeOH in DCM to give **6** (272 mg, off-white solid, 55% from **5**). mp 200–201 °C; *R*_f_ 0.59 (DCM/MeOH 9:1; UV and HBr followed by ninhydrin). [α]_D_^24^ −10.2 (*c* 1.0, MeOH); ^1^H NMR (300 MHz, DMSO-*d*_6_, 25 °C) δ 8.52 (t, ^3^*J*_H,H_ = 5.6 Hz, 1H, N*H*-CH_2_-pyrene), 8.39–8.20 (m, 5H, CH pyrene), 8.16 (s, 2H, CH pyrene), 8.13–7.91 (m, 4H, NH Leu + NH Lys + CH pyrene), 6.75 (t, ^3^*J*_H,H_ = 5.4 Hz, 1H, NH Boc), 5.01 (d, ^3^*J*_H,H_ = 5.7 Hz, 2H, NH-C*H*_2_-pyrene), 4.40–4.19 (m, 2H, α-CH Leu + α-CH Lys), 3.03 (d, ^3^*J*_H,H_ = 5.0 Hz, 1H, α-CH Val), 2.90–2.75 (m, 2H, α-CH_2_ Lys), 1.89–1.77 (m, 1H, β-CH Val), 1.74–1.47 (m, 3H, β-CH_2_ Leu + γ-CH Leu), 1.46–1.15 (m, 15H, *t*Bu + β-CH_2_ Lys + γ-CH_2_ Lys + δ-CH_2_ Lys), 0.88–0.68 (m, 12H, 4 × CH_3_ Val and Leu); ^13^C NMR (75 MHz, CDCl_3_, 25 °C) δ 172.0 (2 × C=O amide), 171.4 (C=O amide), 155.5 (C=O Boc), 132.7 (C quat. pyrene), 130.8 (C quat. pyrene), 130.3 (C quat. pyrene), 130.1 (C quat. pyrene), 128.1 (C quat. pyrene), 127.5 (CH pyrene), 127.4 (CH pyrene), 127.0 (CH pyrene), 126.6 (CH pyrene), 126.3 (CH pyrene), 125.3 (CH pyrene), 125.2 (CH pyrene), 124.7 (CH pyrene), 124.0 (C quat. pyrene), 123.9 (C quat. pyrene), 123.2 (CH pyrene), 77.3 (C quat. *T-*Bu), 59.5 (α-CH Val), 52.7 (α-CH Lys), 50.8 (α-CH Leu), 40.8 (β-CH_2_ Leu), ~39.5 (ε-CH_2_ Lys + NH-*C*H_2_-pyrene buried by DMSO), 31.7 (β-CH_2_ Lys), 31.5 (β-CH Val), 29.2 (CH_2_ Leu), 28.3 (*t-*Bu CH_3_), 24.1 (γ-CH Leu), 23.0 (CH_3_), 22.8 (CH_2_ Leu), 21.4 (CH_3_), 19.4 (CH_3_), 16.9 (CH_3_); IR (KBr) 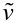: 3275 (w), 3043 (w), 2957 (w), 2930 (w), 2870 (w), 1678 (m), 1627 (s), 1530 (s), 1468 (m), 1390 (m), 1365 (m), 1276 (m), 1250 (m), 1168 (m), 1101 (w), 1064 (w), 1009 (w), 962 (w), 892 (w), 840 (s), 819 (m), 751 (m) cm^−1^; HRMS (ESI^+^) *m*/*z*: [M + H^+^]: calcd for C_39_H_54_N_5_O_5_: 672.4125; found: 672.4128.

**Synthesis of APTES-functionalized magnetite nanoparticles (NP@APTES)** [34]**:** FeCl_2_·4H_2_O (2.5 mmol) and FeCl_3_·6H_2_O (5 mmol) were dissolved in Milli-Q water at pH 2 under N_2_ atmosphere and vigorous mechanical stirring. Once the solution reached 75 °C, a proper amount of NaOH aqueous solution (2 M) was quickly added, causing the sudden appearance of a black precipitate. The reaction was continued for 20 min, after which the particles were washed several times with boiling water and magnetically collected after each wash, in order to reach neutral pH. Finally, a known volume of water was added to disperse ultrafine magnetic particles to a final concentration of 17 g/L.

**Synthesis of conjugated nanoparticles 9:** 28.6 mg of NP@APTES were dispersed in dry DCM (2 mL) under N_2_ atmosphere. Et_3_N (19 μL, 135 μmol) and bis(trichloromethyl)carbonate (triphosgene) (5.4 mg, 18 μmol) were added at 0 °C. The mixture was stirred at rt for 20 min; then the solvent was evaporated and the nanoparticles were dispersed in dry THF (2 mL) under N_2_ atmosphere. DIPEA (15 μL, 86 μmol) and **7** (28.6 mg, 43 μmol) were added. The reaction occurred in oil bath at 50 °C for 18 h. The final material was magnetically washed with EtOH and stored under vacuum.

**(10*S*,13*S*,16*R*)-13-Isobutyl-16-isopropyl-2,2-dimethyl-4,12,15,18-tetraoxo-10-((pyren-1-ylmethyl)carbamoyl)-3-oxa-5,11,14,17-tetraazahexacosan-26-oic acid (12):** A solution of **6** (99 mg, 0.147 mmol) in dry DMF (4 mL, 0.04 M) was treated with DIPEA (128 μL, 0.735 mmol), monomethyl azelate (31 mg, 0.154 mmol) and HATU (56 mg, 0.154 mmol) at rt under N_2_ atmosphere. After stirring at rt for 3 h, the mixture was partitioned between EtOAc (20 mL) and brine (20 mL). Although the desired product was rather insoluble in both phases, it tends to disperse in the organic phase, and thus separation was anyway possible. The aqueous phase was extracted with EtOAc (2 × 20 mL) and the combined organic phases were washed with brine (3×), and directly concentrated to dryness. The residue (yellow solid) was used in the next step without further purification. It was taken up in DMF (4 mL, 0.04 M) and treated with 1 M NaOH (aqueous solution, 300 μL, 0.300 mmol) at rt. After stirring for 5 h, the mixture was partitioned between EtOAc (20 mL) and (NH_4_)H_2_PO_4_ 5% aqueous solution (20 mL) 0.1 N HCl was added until pH 4. Although the desired product was rather insoluble in both phases, it tends to disperse in the organic phase, and thus separation was anyway possible. The aqueous phase was extracted with EtOAc (3 × 10 mL) and the combined organic phases were washed with brine (3×) and directly concentrated to dryness. The residue (yellow solid) was triturated with Et_2_O to give **9** (106 mg, white solid, 85% from **7**). mp 238 °C with decomposition; *R*_f_ 0.24 (DCM/MeOH 95:5; UV and CAM). [α]_D_^24^ 10.7 (*c* 0.49, EtOH); ^1^H NMR (300 MHz, DMSO-*d*_6_, 25 °C) δ 8.44–8.20 (m, 7H, N*H*-CH_2_-pyrene + NH Val + CH pyrene), 8.15 (s, 2H, CH pyrene), 8.07 (t, ^3^*J*_H,H_ = 7.6 Hz, 1H, CH pyrene), 8.00 (d, ^3^*J*_H,H_ = 7.9 Hz, 1H, CH pyrene), 7.94 (d, ^3^*J*_H,H_ = 7.7 Hz, 2H, NH Leu + NH Lys), 6.75 (t, ^3^*J*_H,H_ = 5.6 Hz, 1H, NH Boc), 4.99 (d, ^3^*J*_H,H_ = 5.7 Hz, 2H, CH_2_-pyrene), 4.26–4.13 (m, 2H, α-CH Leu + α-CH Lys), 4.02 (t, ^3^*J*_H,H_ = 7.3 Hz, 1H, α-CH Val), 2.92–2.80 (m, 2H, ε-CH_2_ Lys), 2.12 (t, ^3^*J*_H,H_ = 7.4 Hz, 2H, C*H_2_*CO_2_H), 2.08–1.97 (m, 1H), 1.96–1.82 (m, 2H,), 1.80–1.52 (m, 3H), 1.52–1.42 (m, 2H), 1.36 (s, 9H, *t-*Bu), 1.42–1.15 (m, 8H), 1.15–0.95 (m, 6H), 0.94–0.67 (m, 12H, 4×CH_3_ Val and Leu); ^13^C NMR (75 MHz, DMSO-*d*_6_, 25 °C) δ 174.6 (C=O), 173.0 (C=O), 172.2 (C=O), 172.2 (C=O), 171.5 (C=O), 155.5 (C=O Boc), 132.7 (C quat. pyrene), 130.8 (C quat. pyrene), 130.3 (C quat. pyrene), 130.1 (C quat. pyrene), 127.9 (C quat. pyrene), 127.5 (CH pyrene), 127.4 (CH pyrene), 127.0 (CH pyrene), 126.3 (CH pyrene), 126.2 (CH pyrene), 125.2 (CH pyrene), 125.2 (CH pyrene), 124.7 (CH pyrene), 124.0 (C quat. pyrene), 123.9 (C quat. pyrene), 123.1 (CH pyrene), 77.3 (C quat. *t-*Bu), 58.8 (α-CH Val), 53.3 (α-CH Lys or α-CH Leu), 51.4 (α-CH Lys or α-CH Leu), ≈39.52 (β-CH_2_ Leu + ε-CH_2_ Lys + CH_2_-pyrene buried by DMSO), 34.8 (CH_2_), 33.7 (*C*H_2_CO_2_H), 31.2 (CH_2_), 29.8 (CH), 29.3(CH_2_), 28.5 (3×CH_2_), 28.3 (*t-*Bu CH_3_), 25.1(CH_2_), 24.5 (CH_2_), 24.1 (CH_2_), 23.2 (CH_3_), 23.1 (CH), 20.8 (CH_3_), 19.0 (CH_3_), 18.7 (CH_3_); IR (KBr) 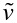: 3272 (m), 3049 (w), 2930 (w), 2869 (w), 1680 (m), 1626 (s), 1532 (s), 1457 (m), 1390 (m), 1366 (m), 1277 (m), 1249 (m), 1226 (m), 1168 (m), 1102 (w), 1011 (w), 961 (w), 914 (w), 841 (m), 820 (w), 752 (m), 704 (m), 680 (m), 654 (m), 619 (m) cm^−1^; HRMS (ESI^+^) *m*/*z* [M + H^+^]: calcd for C_48_H_68_N_5_O_8_: 842.5068; found: 842.5074.

**Synthesis of conjugated nanoparticles 13:** 30 mg of NP@APTES were dispersed in dry DMF (1 mL) under N_2_ atmosphere. **12** (30 mg, 0.036 mmol), DIPEA (31 μL, 0.178 mmol) and HATU (14 mg, 0.037 mmol) were added. The mixture was mechanically stirred vigorously for 18 h at rt. The final material was magnetically washed with EtOH and stored under vacuum.

**Enzymatic reaction on the model compound:** A solution of **6** (13 mg, 0.0198 mmol) in dry DCM/TFA 20:1 (2.0 mL, 0.01 M) was stirred at rt for 2 h. After removal of the volatile components, the residue was taken up with *n*-heptane (×3) and the solvent was evaporated again to give **7** as an off-white solid that was quantitatively transferred to a 10 mL graduated flask with MeOH obtaining a 1.98 mM stock solution of **7**. TRIS buffer (pH 7.5) was freshly prepared by dissolving 3.64 g of TRIS in 50 mL of deionized water and subsequent addition of 1N HCl until pH 7.5. The volume was adjusted to 100 mL in a volumetric flask with deionized water. 0.3 U/mL stock solution of plasmin from human plasma (Sigma-Aldrich P1867-150 μg) was prepared by dissolving 150 μg of lyophilized powder in 1 mL of TRIS buffer. 0.1 mg/mL stock solutions of trypsin from porcine pancreas (Sigma-Aldrich T4799) were prepared by dissolving 5 mg of enzyme in 50 mL of TRIS buffer. **7** (25 μL of stock solution, 50 nmol), plasmin (77 μL of stock solution, 0.023 U) and 730 μL of TRIS buffer were added in a 2 mL Eppendorf. **7** (25 μL of stock solution, 50 nmol), trypsin (46 μL of stock solution, 4.6 μg, 92 μg/μmol) and 760 μL of TRIS buffer were added in a 2 mL Eppendorf. Each enzymatic reaction was carried out at 37 °C in thermomixer (650 rpm) and was monitored after 24 h and 48 h by HPLC-FLD. For the reaction with plasmin, the observed conversions were 88.8% and 93.8% at 24 and 48 h, respectively. With trypsin, the observed conversions were 96.7% and 98.0% at 24 and 48 h, respectively. HPLC conditions. Column: C6 Phenyl 150 × 3 mm, 3 μ. Temp. 25 °C. (H_2_O + 0.1% TFA)/CH_3_CN 95:5 to 41:59 in 20 min. Detection: λ_max_ Ex: 273 nm; λ_max_ Em: 392 nm. *R*_t_ 18.6 min (**7**), 19.9 min (pyrenylmethylamine). From these experiments we deduced that 1 U of plasmin has an activity approximatively similar to 150 μg of trypsin and that complete cleavage of the linker from **7** was achieved in 48 h using 92 μg/μmol of trypsin.

**Enzymatic cleavage of pyrenylmethylamine from conjugated nanoparticles 9:** The enzymatic cleavage is preceded by the cleavage of Boc. In a vial containing **9** (10 mg, corresponding to 0.79 μmol) a solution of dry DCM/TFA 20:1 (200 μL) was added. The reaction was run for 4 h under vigorous shaking. The sample was then dried and used for the enzymatic cleavage without any further purification. In an Eppendorf vial containing deprotected **9**, 975 μL of a 0.1 mg/mL trypsin stock solution (corresponding to 123 μg/μmol) were added. The final volume was adjusted to 1 mL with TRIS buffer. The sample was kept under shaking in a thermomixer (650 rpm) at 37 °C for 72 h. The sample was then washed several times with MeOH using both magnetic washing and centrifugation (Eppendorf 15,000 rpm 10 min each) recovering the washings in a volumetric 10 mL flask. The sample, before being injected in the HPLC-VWD, was preconcentrated by a factor of 20 (thus to 500 μL). The quantitative determination of 1-pyrenylmethylamine was carried out through a calibration curve (see Supporting Information File 1), and resulted in 26 μg/mL = 13.0 μg (56.1 nmol). The percent of pyrenylmethylamine released is thus 7.1%. HPLC conditions. Column: C6 Phenyl 150 × 3 mm, 3 μ. Temp. 25 °C. Injected volume: exactly 5 μL. Eluents: (H_2_O + 0.1% TFA)/CH_3_CN 95:5 to 41:59 in 20 min. Detection: 240 nm. *R*_t_ = 19.9 min.

**Enzymatic cleavage of pyrenylmethylamine from conjugated nanoparticles 13:** The enzymatic cleavage is preceded by the cleavage of Boc. In a vial containing **13** (20 mg, corresponding to 3.68 μmol) a solution of dry DCM/TFA 20:1 (400 μL) was added. The reaction was run for 4 h under vigorous shaking. The sample was then dried and used for the enzymatic cleavage without any further purification. In an Eppendorf vial containing deprotected **13**, 920 μL of a 0.5 mg/mL trypsin stock solution (corresponding to 136 μg/μmol) were added. The final volume was adjusted to 1 mL with TRIS buffer. The sample was kept under shaking in a thermomixer (650 rpm) at 37 °C for 72 h. The sample was then washed several times with MeOH using both magnetic washing and centrifugation (Eppendorf 15,000 rpm 10 min each) recovering the washings in a volumetric 10 mL flask. The sample, before being injected in the HPLC-VWD, was preconcentrated by a factor of 20 (thus to 500 μL). The quantitative determination of 1-pyrenylmethylamine was carried out through a calibration curve (see Supporting Information File 1), and resulted in 98.8 μg/mL = 49.4 μg (213 nmol). The sample injected in the HPLC-VWD was preconcentrated by a factor of 20. The percent of pyrenylmethylamine released is thus 5.8%. The HPLC conditions are as given above.

## Supporting Information

File 1 Additional experiments and NMR spectra of all new compounds.
Details: Diameter distribution function of NP@APTES and NP@silica@APTES obtained from DLS measurements; optimization of the coupling of **5** with a model amine and of allyl urethane cleavage; calibration curve for pyrenylmethylamine; ^1^H and ^13^C spectra of all new compounds.
