# Supplementary material for: Enzymatically promoted release of organic molecules linked to magnetic nanoparticles
Source: Beilstein J Nanotechnol. 2018 Mar 27;9:986–99. doi: 10.3762/bjnano.9.92 (PMC5905276; doi:10.3762/bjnano.9.92)
Supplement: File 1 — Additional experiments and NMR spectra of all new compounds. Details: Diameter distribution function of NP@APTES and NP@silica@APTES obtained from DLS measurements; optimization of the coupling of 5 with a model amine and of allyl urethane cleavage; calibration curve for pyrenylmethylamine; 1H and 13C spectra of all new compounds. [file Beilstein_J_Nanotechnol-09-986-s001.pdf]

Supporting Information

for

Enzymatically promoted release of organic molecules linked to magnetic nanoparticles

Chiara Lambruschini[†1], Silvia Villa[†1], Luca Banfi[1], Fabio Canepa[1], Fabio Morana[1], Annalisa Relini[1], Paola Riani*[1], Renata Riva*[1] and Fulvio Silvetti[1]

Address: Department of Chemistry and Industrial Chemistry, Università di Genova, via Dodecaneso, 31 16146 Genova, Italy

Email: Paola Riani - paola.riani@unige.it; Renata Riva - renata.riva@unige.it

* Corresponding author

[†] Contributed equally to this work

Additional experiments and NMR spectra of all new compounds

Index:

- Diameter distribution function of NP@APTES and NP@SILICA@APTES obtained from DLS measurements
- Optimization of the coupling of **5** with a model amine
- Optimization of allyl urethane cleavage
- Calibration curve for pyrenylmethylamine
- ^{1}H and ^{13}C spectra of all new compounds

Diameter distribution function of NP@APTES and NP@SILICA@APTES obtained from DLS measurements

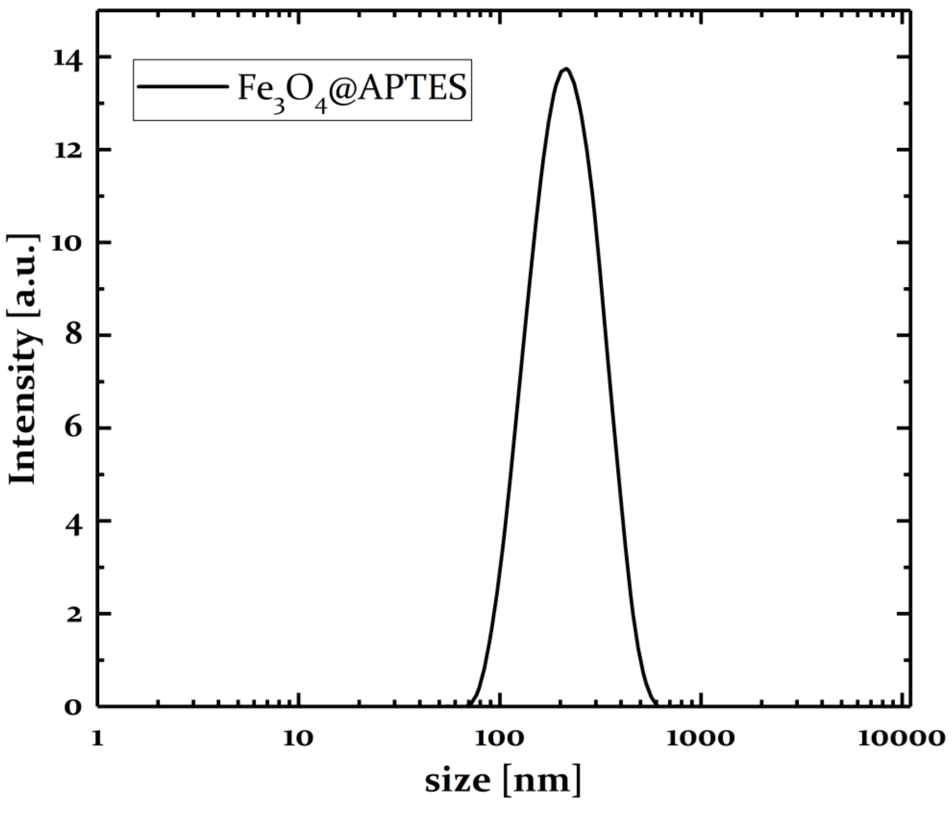

Figure S1: Size distribution of Fe_3O_4@APTES NPs by DLS technique.

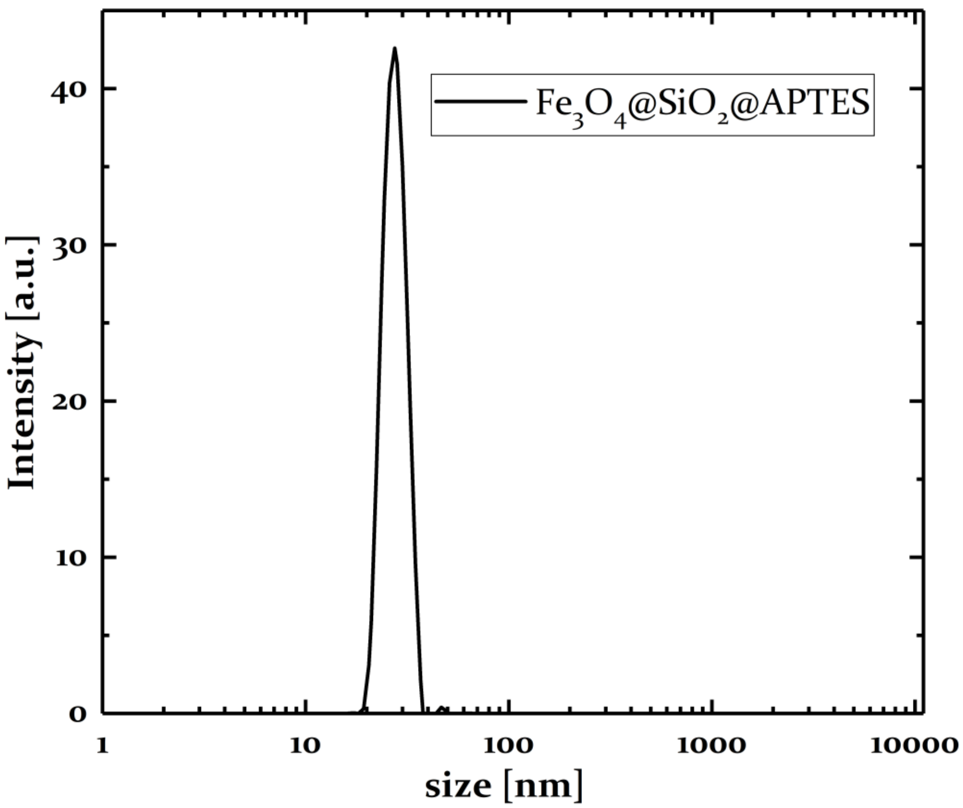

Figure S2: Size distribution of Fe_3O_4@SiO_2@APTES by the DLS technique.

Optimization of coupling of 5 with a model amine

This coupling was optimized using benzylamine as model compound. The diastereomeric ratios were determined by HPLC, and the epimer of the desired product was recognized by performing HPLC-MS analysis.

The following Table reports the obtained results. The reactions were all carried out at rt, using 1 equivalent of coupling agent and 5 equivalent of base.

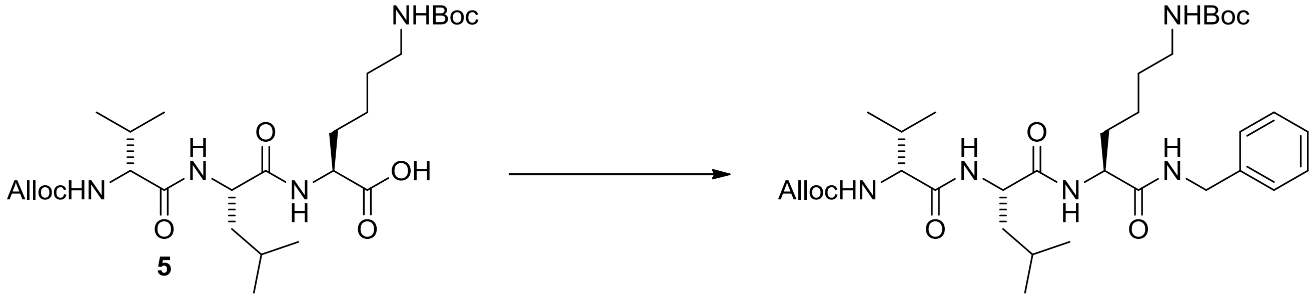

Coupling agent	Base	Solvent	Diast. ratio
PyBOP	NMM	CH_2Cl_2	67 : 33
EDC-HOBT	DIPEA	CH_2Cl_2	69 : 31
HBTU	DIPEA	CH_2Cl_2	90 : 10
TBTU	DIPEA	CH_2Cl_2	92 : 8
HATU	DIPEA	CH_2Cl_2	96 : 4
HATU	*sym*-collidine	DMF	93 : 7
HATU	NMM	DMF	95 : 5
HATU	DIPEA	DMF	97.5 : 2.5

Abbreviations: PyBOP = (Benzotriazol-1-yl-oxytripyrrolidinophosphonium hexafluorophosphate). EDC = 1-Ethyl-3-(3-dimetilaminopropyl)carbodiimide. HBTU = 3-[Bis(dimethylamino)methylene]-3H-benzotriazol-1-oxid hexafluorophosphate. TBTU = 3-[Bis(dimethylamino)methylene]-3H-benzotriazol-1-oxid tetrafluoroborate. HOBT: 1-hydroxybenzotriazole. DIPEA: *N*,*N*,-diisopropylethylamine (DPEA). HATU: 1-[bis(dimethylamino)methylene]-1H-1,2,3-triazolo[4,5-b]pyridinium 3-oxid hexafluorophosphate. NMM = *N*-Methylmorpholine.

Optimization of allyl urethane cleavage

This deblocking was optimized using model compound **A**, which was prepared from **5** in 91% yield by treatment with benzylbromide and K_2CO_3 in DMF for 1.5 h at rt.

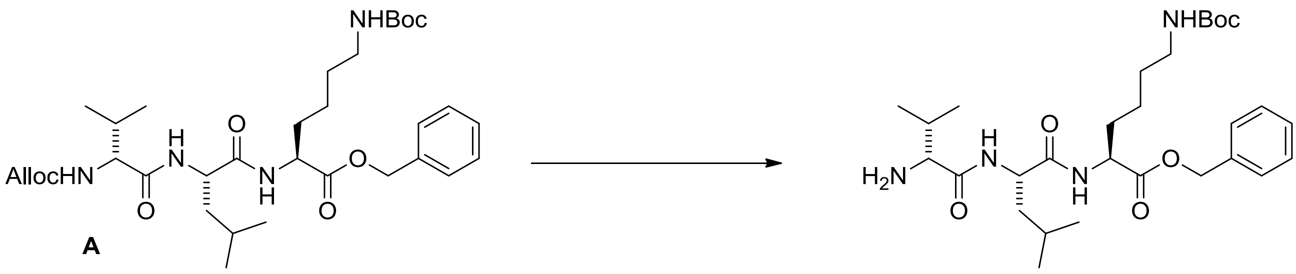

Catalyst	Scavenger	Other conditions	Yield
$Pd(PPh_3)_4$ (0.20 equiv.)	pyrrolidine (10 equiv.)	CH_2Cl_2, rt	19%
$Pd(PPh_3)_4$ (0.20 equiv.)	thioanisole (10 equiv.)	CH_2Cl_2, rt	11%
$Pd(PPh_3)_4$ (0.20 equiv.)	phenylsilane (10 equiv.)	CH_2Cl_2, rt	22%
$Pd(PPh_3)_4$ (0.10 equiv.)	pyrrolidine (5 equiv.)	THF, rt	<10%
$Pd(PPh_3)_4$ (0.10 equiv.)	phenylsilane (5 equiv.)	THF, rt	75%
$Pd(PPh_3)_4$ (0.10 equiv.)	dimedone (5 equiv.)	THF, rt	60%
$Pd(PPh_3)_4$ (0.10 equiv.)	triethylammonium formate (5 equiv.)	THF, rt	42%
$Pd(PPh_3)_4$ (0.10 equiv.)	phenylsilane (10 equiv.)	THF, rt	90%

Calibration curve for pyrenylmethylamine

The quantitative determination of 1-pyrenylmethylamine was carried out through a calibration curve, by injecting 5 μL of solutions of known concentration.

HPLC conditions. Column: C6 Phenyl 150 × 3 mm, 3 μ. Temp. = 25 °C. (H_2O + 0.1% TFA)/CH_3CN 95:5 to 41:59 in 20 min. Detection: 240 nm. R_t = 19.9.

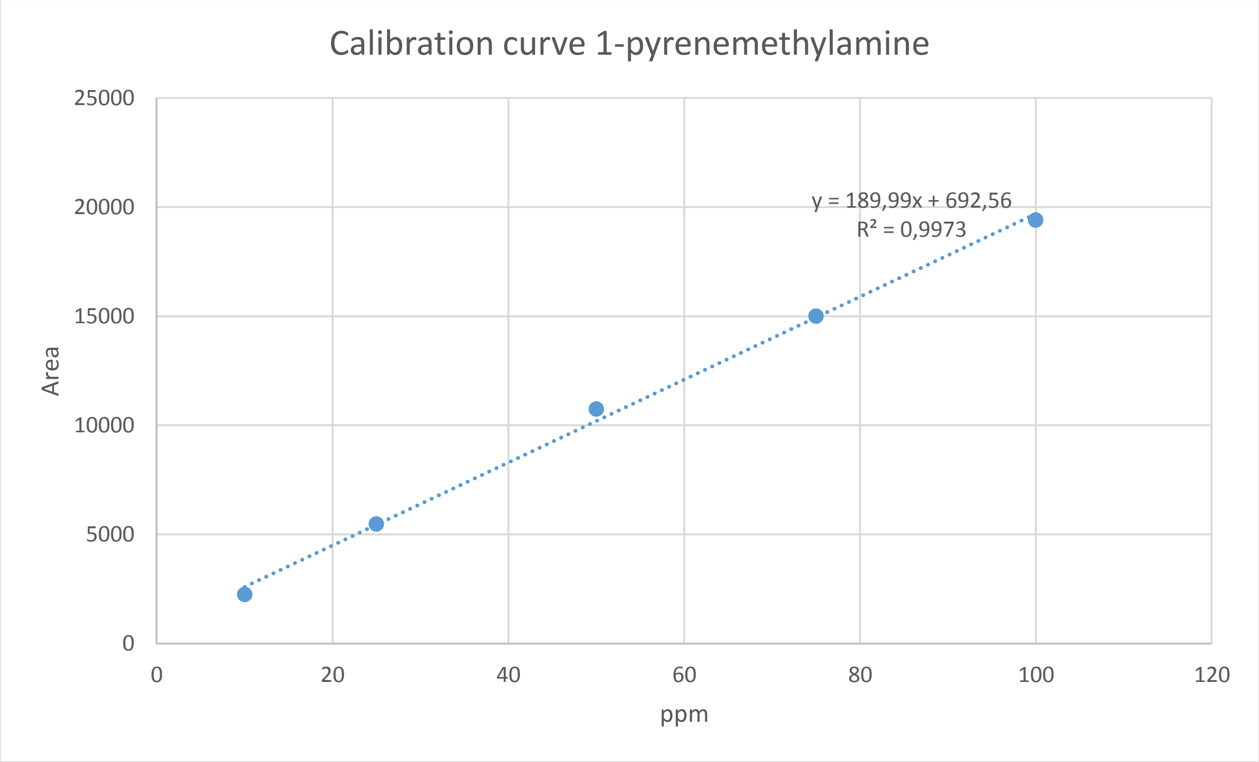

* ppm corresponds to μg/mL.

^{1}H and ^{13}C spectra of all new compounds

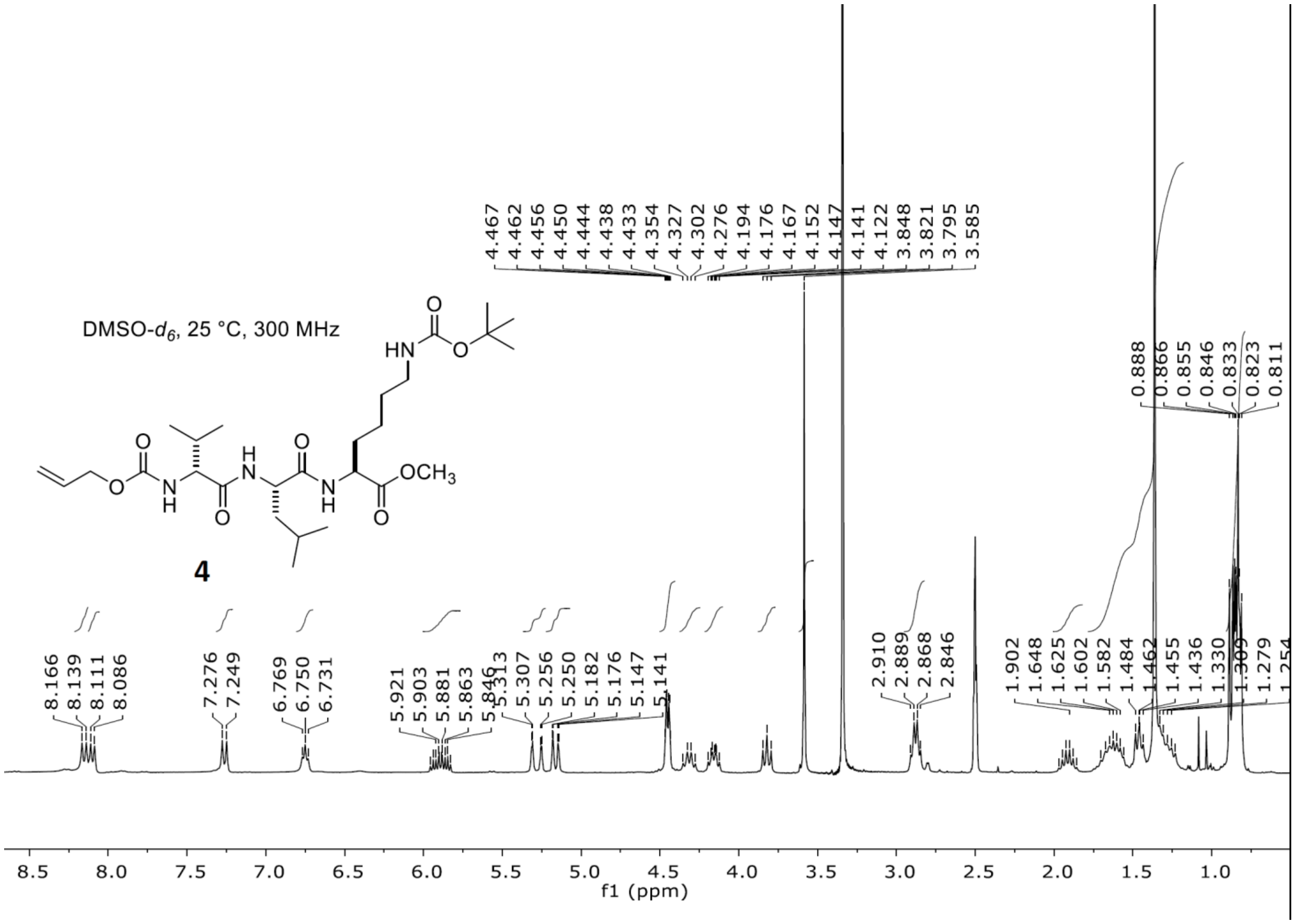

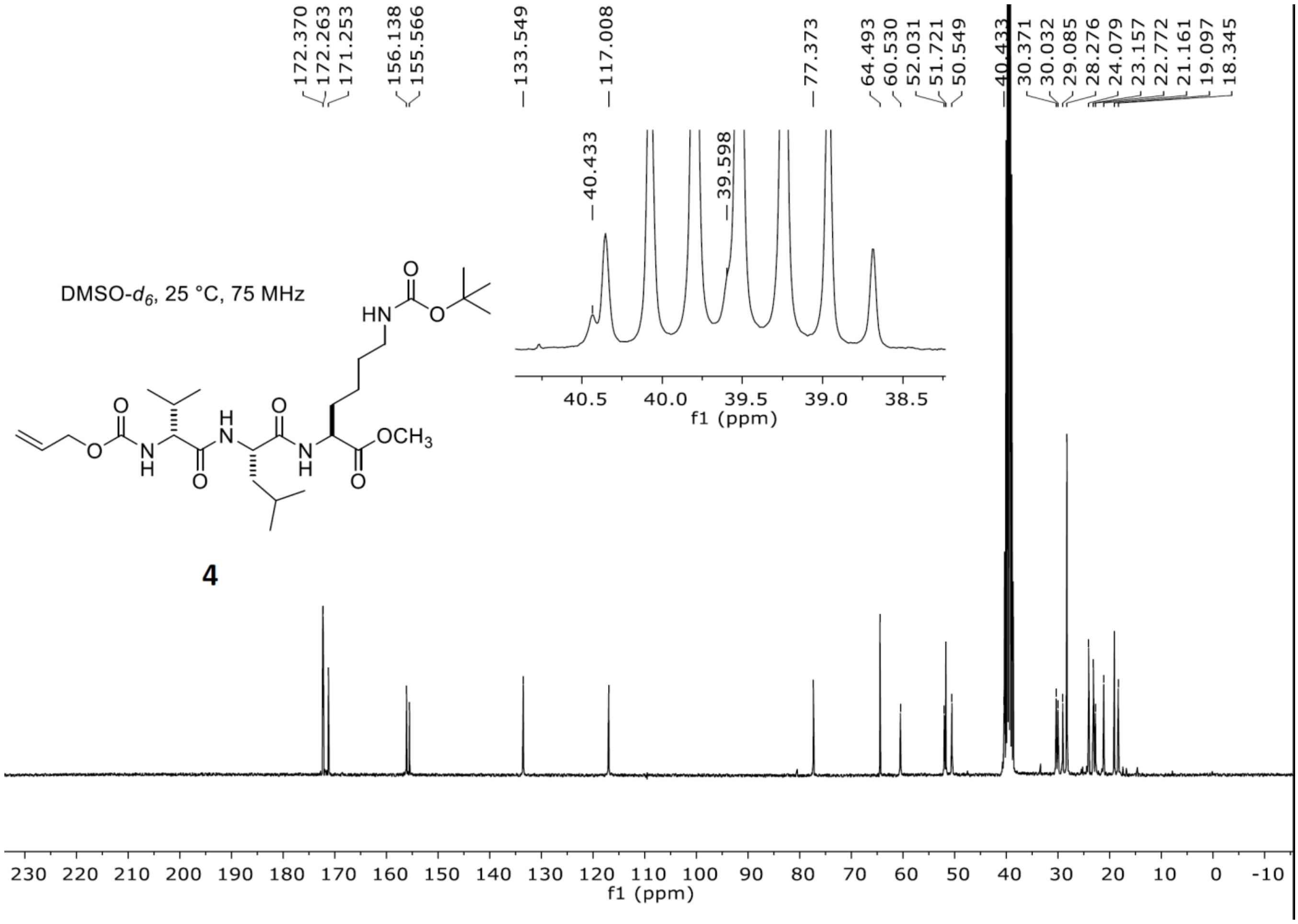

DMSO-d_6, 25 °C, 75 MHz
4
172.370
172.263
171.253
156.138
155.566
133.549
117.008
77.373
64.493
60.530
52.031
51.721
50.549
40.433
30.371
30.032
29.085
28.276
24.079
23.157
22.772
21.161
19.097
18.345
40.433
39.598
40.5 40.0 39.5 39.0 38.5
f1 (ppm)
230 220 210 200 190 180 170 160 150 140 130 120 110 100 90 80 70 60 50 40 30 20 10 0 -10
f1 (ppm)

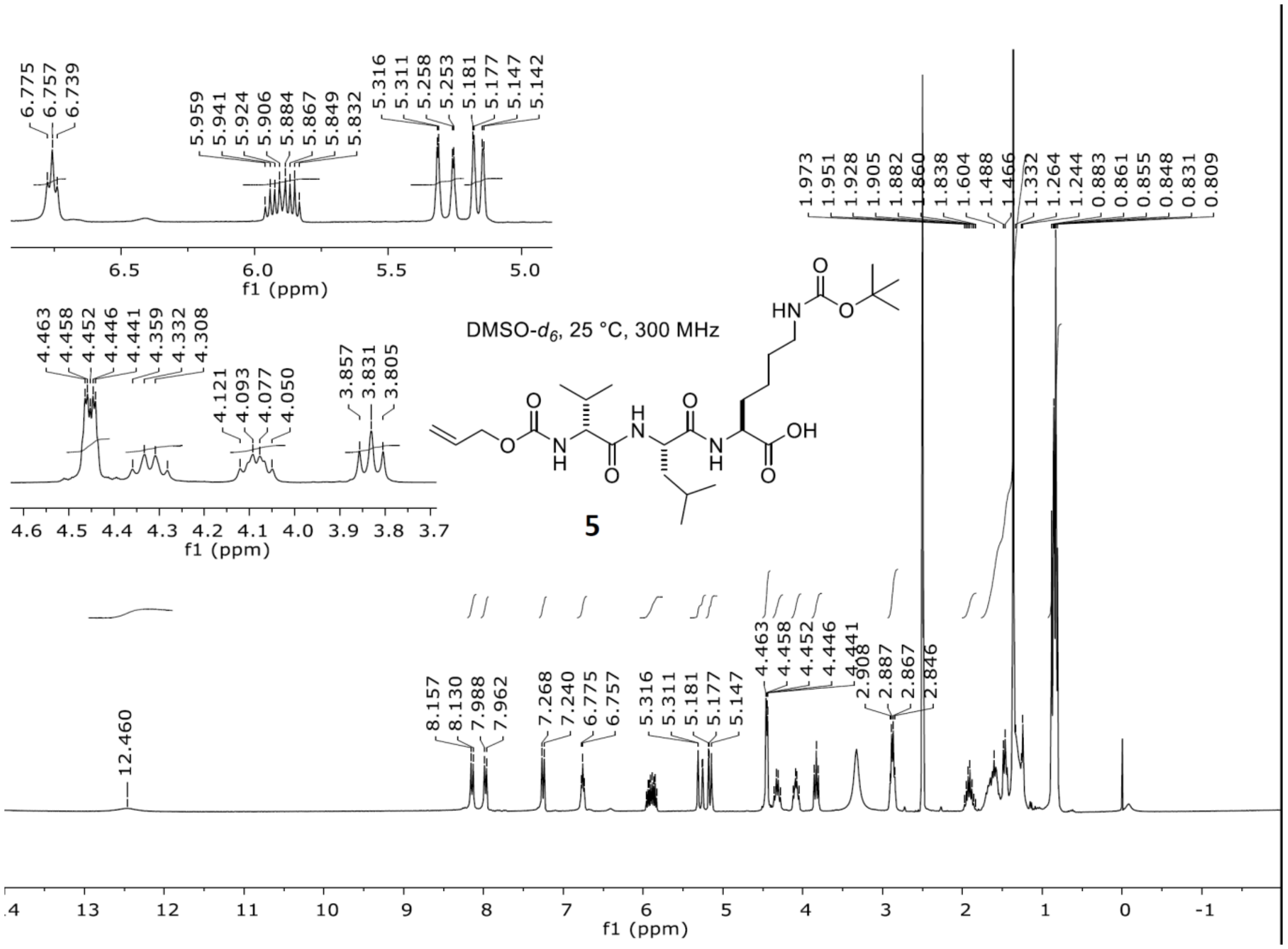
DMSO-d_6, 25 °C, 300 MHz
5
HN
O
O
N
H
OH
6.775 6.757 6.739
5.959 5.941 5.924 5.906 5.884 5.867 5.849 5.832
5.316 5.311 5.258 5.253 5.181 5.177 5.147 5.142
4.463 4.458 4.452 4.446 4.441 4.359 4.332 4.308
4.121 4.093 4.077 4.050
3.857 3.831 3.805
1.973 1.951 1.928 1.905 1.882 1.860 1.838 1.604 1.488 1.466 1.332 1.264 1.244 0.883 0.861 0.855 0.848 0.831 0.809
12.460
8.157 8.130 7.988 7.962
7.268 7.240 6.775 6.757
5.316 5.311 5.181 5.177 5.147
4.463 4.458 4.452 4.446 4.441
2.908 2.887 2.867 2.846
f1 (ppm)

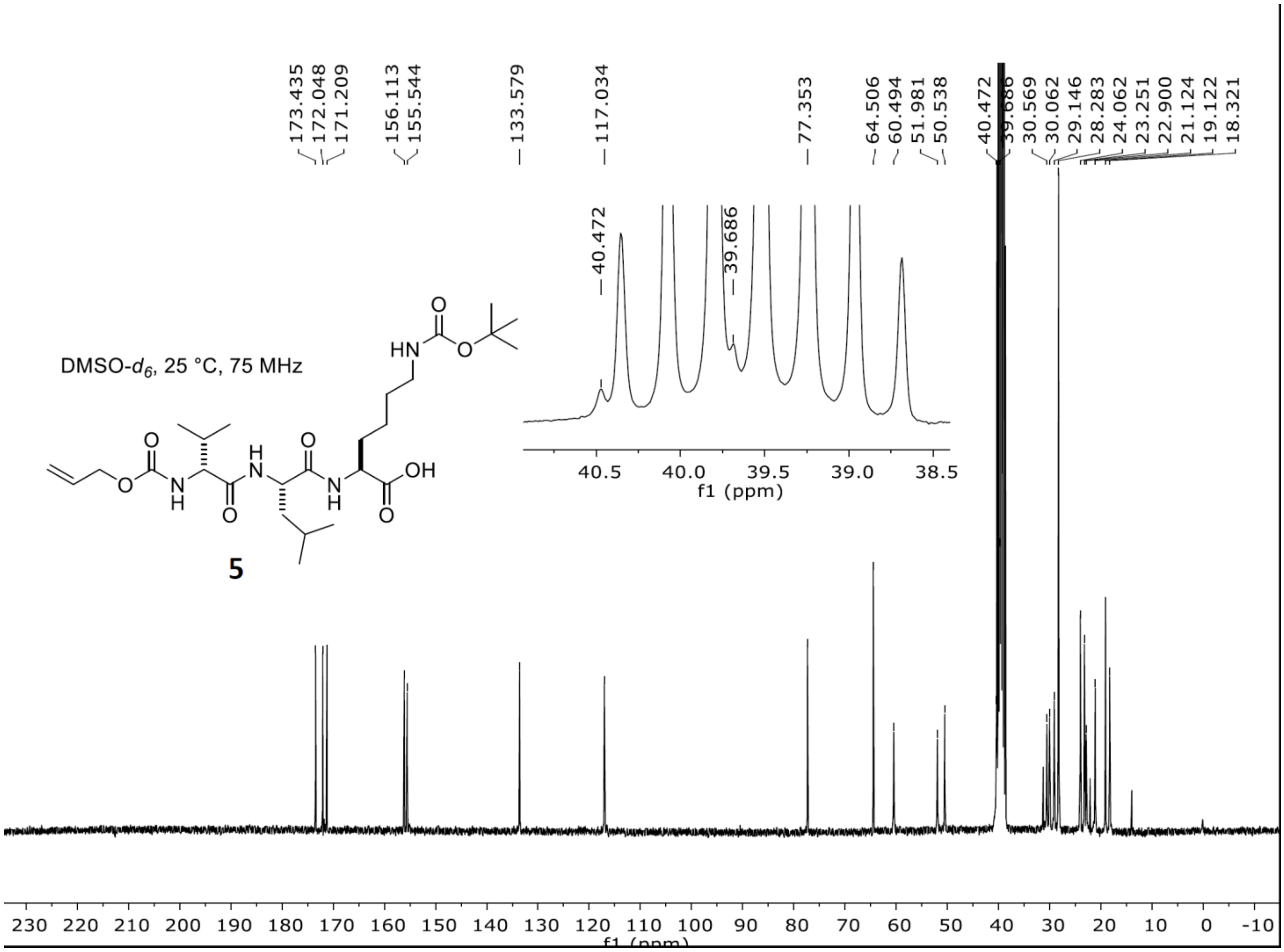
DMSO-d_6, 25 °C, 75 MHz
5
173.435
172.048
171.209
156.113
155.544
133.579
117.034
77.353
64.506
60.494
51.981
50.538
40.472
39.686
30.569
30.062
29.146
28.283
24.062
23.251
22.900
21.124
19.122
18.321
f1 (ppm)

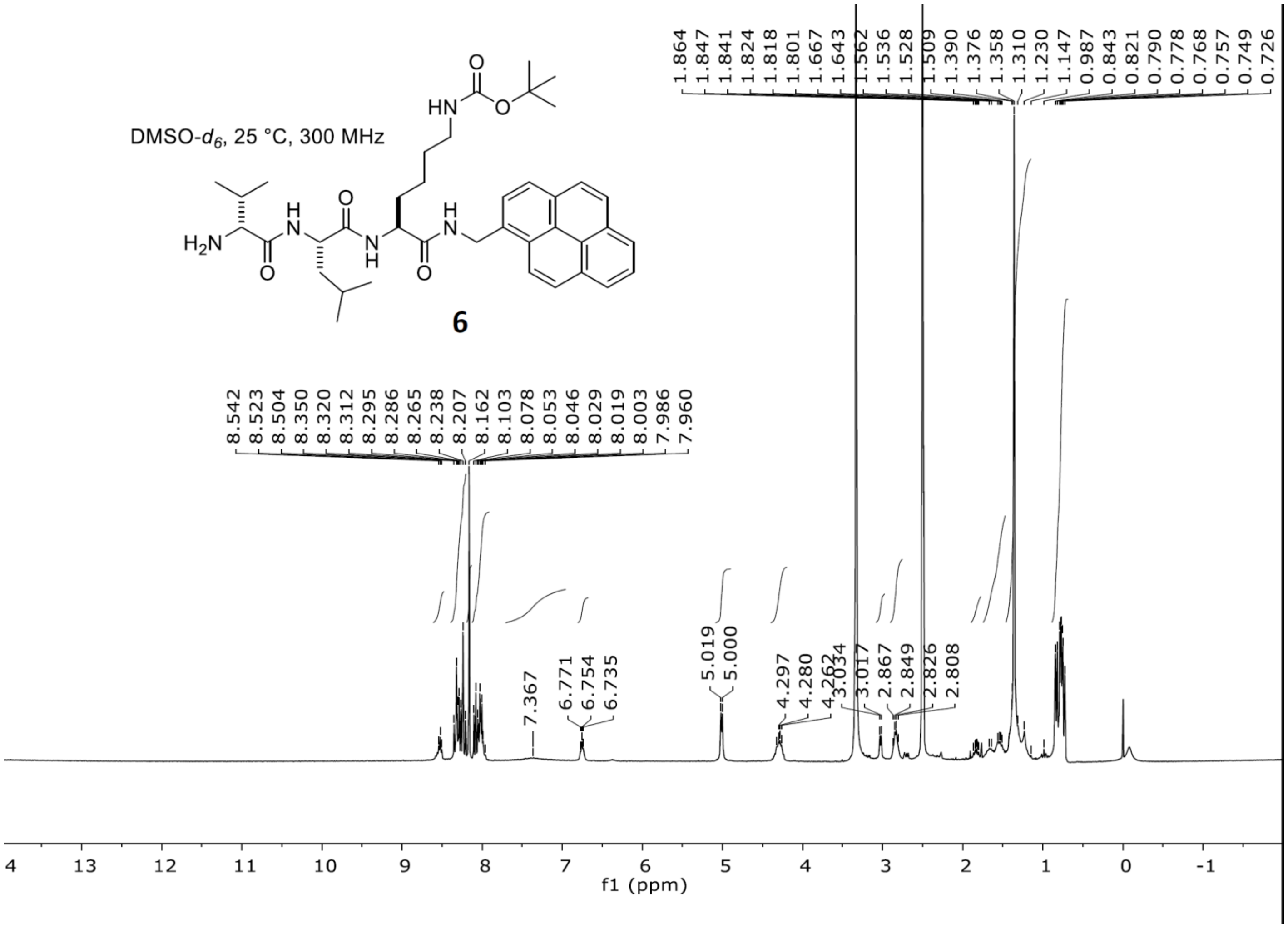
DMSO-d_6, 25 °C, 300 MHz
6
f1 (ppm)

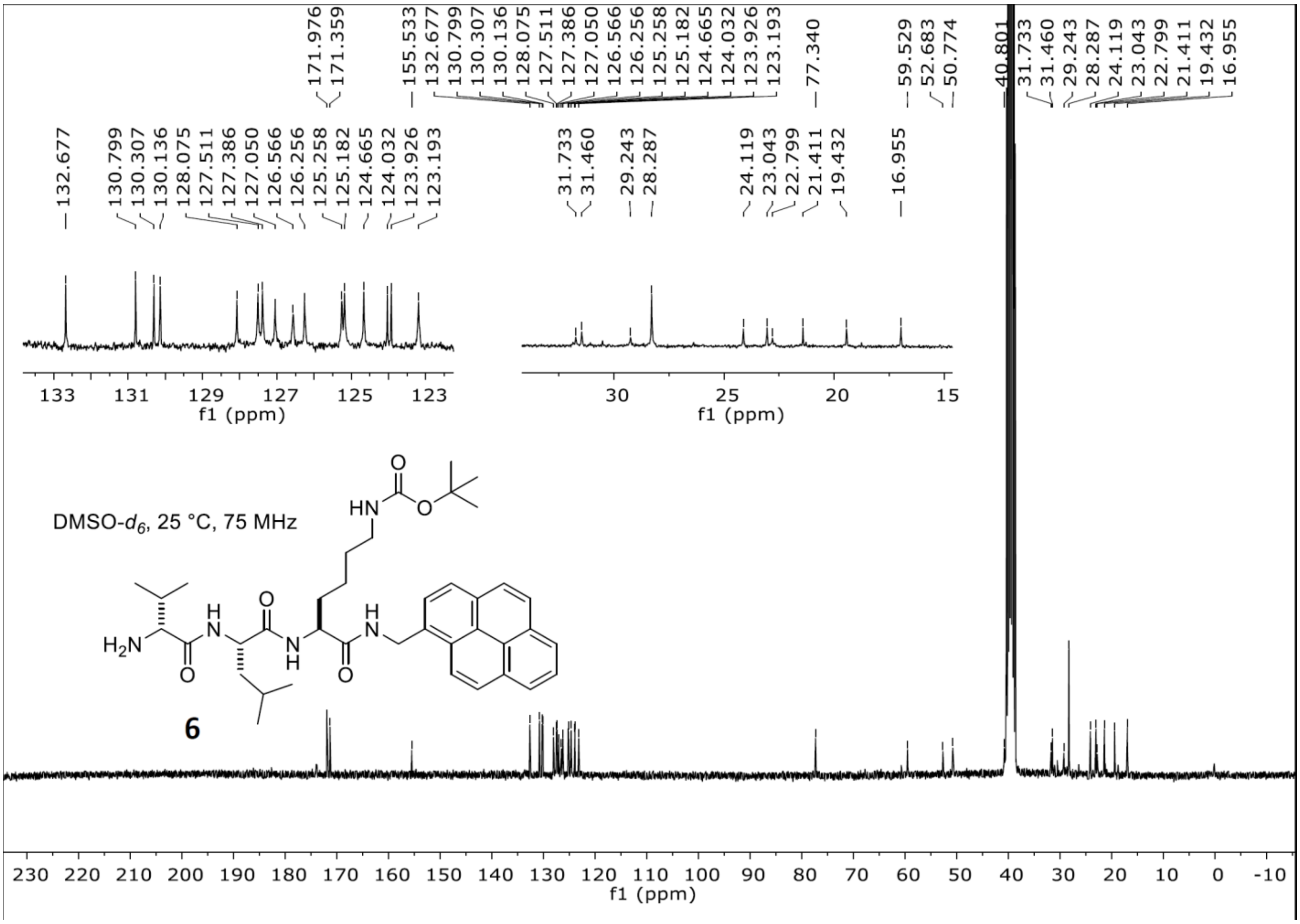
171.976
171.359
155.533
132.677
130.799
130.307
130.136
128.075
127.511
127.386
127.050
126.566
126.256
125.258
125.182
124.665
124.032
123.926
123.193
77.340
59.529
52.683
50.774
40.801
31.733
31.460
29.243
28.287
24.119
23.043
22.799
21.411
19.432
16.955
f1 (ppm)
DMSO-d_6, 25 °C, 75 MHz
6

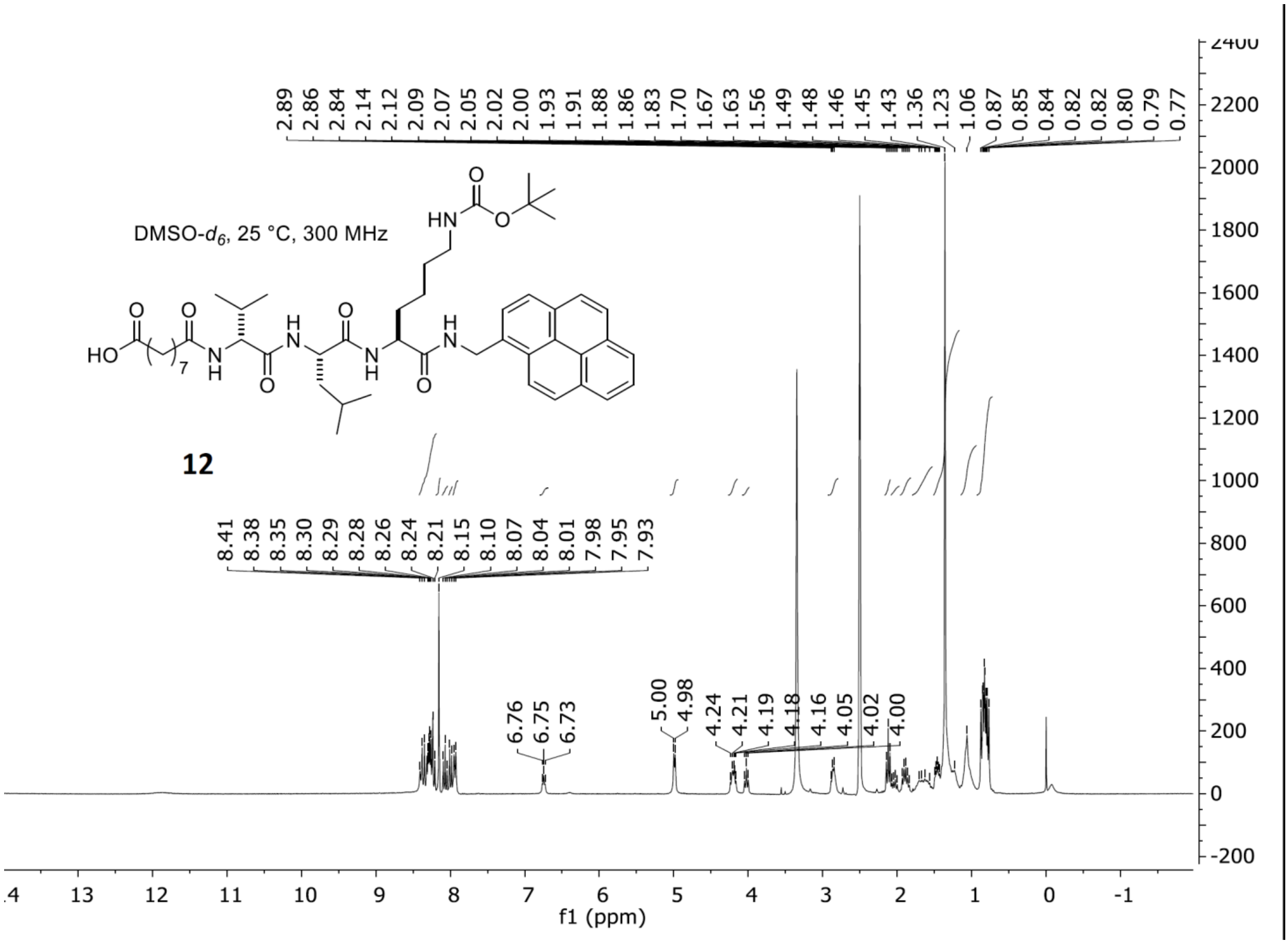
DMSO-d_6, 25 °C, 300 MHz
12
f1 (ppm)

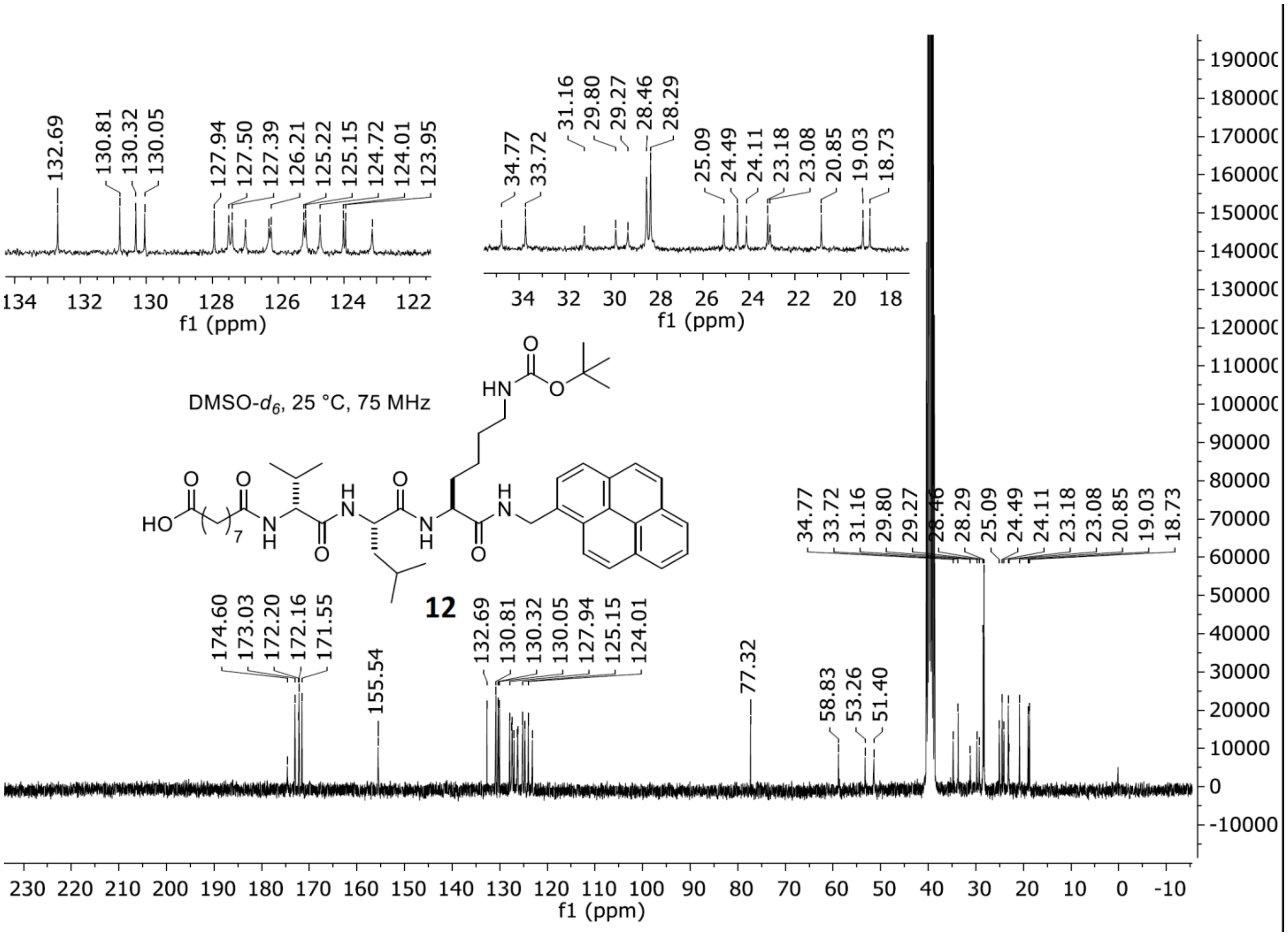
DMSO-d_6, 25 °C, 75 MHz
12
f1 (ppm)
174.60
173.03
172.20
172.16
171.55
155.54
132.69
130.81
130.32
130.05
127.94
127.50
127.39
126.21
125.22
125.15
124.72
124.01
123.95
77.32
58.83
53.26
51.40
34.77
33.72
31.16
29.80
29.27
28.46
28.29
25.09
24.49
24.11
23.18
23.08
20.85
19.03
18.73
